# Source-Modeling Auditory Processes of EEG Data Using EEGLAB and Brainstorm

**DOI:** 10.3389/fnins.2018.00309

**Published:** 2018-05-08

**Authors:** Maren Stropahl, Anna-Katharina R. Bauer, Stefan Debener, Martin G. Bleichner

**Affiliations:** ^1^Neuropsychology Lab, Department of Psychology, European Medical School, University of Oldenburg, Oldenburg, Germany; ^2^Cluster of Excellence Hearing4all, University of Oldenburg, Oldenburg, Germany

**Keywords:** EEG, source localization, Brainstorm, EEGLAB, auditory N100, auditory processing

## Abstract

Electroencephalography (EEG) source localization approaches are often used to disentangle the spatial patterns mixed up in scalp EEG recordings. However, approaches differ substantially between experiments, may be strongly parameter-dependent, and results are not necessarily meaningful. In this paper we provide a pipeline for EEG source estimation, from raw EEG data pre-processing using EEGLAB functions up to source-level analysis as implemented in Brainstorm. The pipeline is tested using a data set of 10 individuals performing an auditory attention task. The analysis approach estimates sources of 64-channel EEG data without the prerequisite of individual anatomies or individually digitized sensor positions. First, we show advanced EEG pre-processing using EEGLAB, which includes artifact attenuation using independent component analysis (ICA). ICA is a linear decomposition technique that aims to reveal the underlying statistical sources of mixed signals and is further a powerful tool to attenuate stereotypical artifacts (e.g., eye movements or heartbeat). Data submitted to ICA are pre-processed to facilitate good-quality decompositions. Aiming toward an objective approach on component identification, the semi-automatic CORRMAP algorithm is applied for the identification of components representing prominent and stereotypic artifacts. Second, we present a step-wise approach to estimate active sources of auditory cortex event-related processing, on a single subject level. The presented approach assumes that no individual anatomy is available and therefore the default anatomy ICBM152, as implemented in Brainstorm, is used for all individuals. Individual noise modeling in this dataset is based on the pre-stimulus baseline period. For EEG source modeling we use the OpenMEEG algorithm as the underlying forward model based on the symmetric Boundary Element Method (BEM). We then apply the method of dynamical statistical parametric mapping (dSPM) to obtain physiologically plausible EEG source estimates. Finally, we show how to perform group level analysis in the time domain on anatomically defined regions of interest (auditory scout). The proposed pipeline needs to be tailored to the specific datasets and paradigms. However, the straightforward combination of EEGLAB and Brainstorm analysis tools may be of interest to others performing EEG source localization.

## Introduction

Despite strong competition from other imaging techniques, the scalp-recorded electroencephalogram (EEG) is still one of the key sources of information for scientists interested in the study of large-scale human brain function. Due to its high temporal resolution EEG acquisition technology is well suited to capture the essence of neural dynamics of perceptual, cognitive and motor processes. However, complex cognitive operations go hand in hand with complex spatio-temporal neuronal interactions. Even for the processing of very simple sounds several brain areas are involved and information of different brain areas has to be incorporated within tens of millisecond (Shahin et al., 2007). Due to volume conduction (among other reasons) the EEG signal recorded from a single channel is a mixture of contributions from an unknown number of different, even distant neural and non-neural sources (Lopes da Silva, 2013). Consequently, differences between conditions or individuals cannot easily be interpreted with regard to their spatial origin when only sensor level data is considered. Source modeling on the other hand allows to draw inferences about the timing and the location of brain processes of interest and may resolve to some degree the ambiguity we are faced with sensor level analysis (Michel et al., 2004; Lopes da Silva, 2013; Baillet, 2017). Despite the fact that a source level analysis does not solve the inverse problem (Musha and Okamoto, 1999; Grech et al., 2008), high-density EEG in combination with source modeling is considered as an electrical brain imaging tool (Michel and Murray, 2012), which helps to confirm predictions about the likely spatial origin of EEG sensor level features. For instance, we have used source level analysis of 96-channel EEG recordings to study cross-modal processing in the auditory cortex of cochlear implant users (Stropahl et al., 2015; Stropahl and Debener, 2017), and showed that in these individuals, auditory cortex is recruited for the processing of visual stimuli. This pattern has been repeatedly confirmed with EEG source analysis, as well as imaging modalities such as functional near infrared spectroscopy (fNIRS), but could not be easily obtained with functional magnetic resonance imaging (fMRI), which cannot be used for cochlear implant users (Sandmann et al., 2012; Stropahl et al., 2015, 2017; Chen et al., 2016). In other studies we used source level analysis to disentangle left and right auditory cortex activation patterns (Hine and Debener, 2007; Hine et al., 2008; Sandmann et al., 2015) and investigate the temporal evolution of auditory entrainment using 64-channel EEG recordings (Bauer et al., 2018).

There are numerous other examples of successful EEG source modeling. Most researchers agree that volume conduction heavily compromises the validity of sensor level connectivity pattern results (Schoffelen and Gross, 2009). Source modeling can facilitate the analysis by mitigating to some degree disadvantageous effects of volume conduction. Hence, source modeling seems useful for studying resting state EEG (Hipp et al., 2012), spontaneous neural oscillations and their spatial origin (Srinivasan et al., 2006) or to disentangle sub-processes of auditory perception (De Santis et al., 2007; Shahin et al., 2007). In a clinical context, EEG source modeling can be used to identify the epileptic focus in epilepsy patients (Brodbeck et al., 2011).

In the context of magnetoencephalography (MEG) analysis source modeling is well established and widely used (Baillet, 2017). There is an ongoing and recurrent debate on the spatial acuity of EEG and MEG source modeling (Cohen and Cuffin, 1991; Crease, 1991; Barkley, 2004; Baumgartner, 2004; Baillet, 2017). MEG and EEG source modeling do not necessarily yield the same source locations (Scheler et al., 2007), as the sensors have different sensitivities to different sources. EEG source modeling appears to be more sensitive to errors in the forward model (Leahy et al., 1998), but with a sufficient number of sensors, and use of an accurate individual head model with reasonable conductivity values, EEG source localization accuracy may be at par with MEG localization accuracy (Malmivuo, 2012; Klamer et al., 2014).

In the following we will show the joint use of two highly popular open source Matlab toolboxes, EEGLAB (Delorme and Makeig, 2004) and Brainstorm (Tadel et al., 2011) for performing EEG source modeling. While the former has been developed primarily for multi-channel EEG analysis, it provides some capabilities for MEG analysis as well. The opposite is true for Brainstorm, which has been designed for MEG analysis but is also well suited for EEG source modeling.

EEGLAB may currently be the most popular EEG analysis toolbox. Each release initiates thousands of downloads, the reference paper has been cited over 5,000 times, and an increasing number of powerful plugins has expanded its functionality. One reason for the popularity of EEGLAB may be that it offers functionality for Matlab newbies (graphical user interface) and fluent programmers alike. Another reason is that EEGLAB facilitates the use of independent component analysis (ICA), a linear decomposition approach, that, if applied correctly (Winkler et al., 2015), performs very well in the attenuation of various EEG artifacts (Jung et al., 2000a). Denoising EEG signals is not the only virtue of ICA, it can also be used to disentangle otherwise missed contributions from different brain sources (Debener et al., 2010). The focus of EEGLAB lies on sensor level analysis and the modeling of statistical, but not bio-physiological sources. The possibilities of performing and consequently visualizing the results of a dipole analysis are limited with EEGLAB.

Brainstorm on the other hand provides extensive possibilities of source estimation and advanced source level analysis on both, single subject and group level. However, the primary focus of brainstorm is on MEG processing, and not all processing steps are optimal or necessary for EEG data, and may not be overly intuitive for EEGLAB users. Furthermore, source modeling will often be only one step in the signal analysis of an otherwise complete analysis that could be performed with EEGLAB and custom-made Matlab routines.

Here, we demonstrate how a well-established EEGLAB based pre-processing (including ICA artifact attenuation) sensor level analysis can be combined with the source modeling of this pre-processed EEGLAB data in Brainstorm. We present a pipeline for computing single subject as well as group level source activity for EEG data when no individual anatomical data is available, using a standard head model as implemented in Brainstorm. The pipeline provides an easy way to estimate and compare source activity in (pre-defined) regions of interest.

## Materials and equipment

### Participants

Data was collected from 10 participants with a mean age of μ = 48 years (*SD* = 13.7 years, age range: 21–68 years; 7 females, 3 males). All participants had normal or corrected-to-normal vision and normal hearing (thresholds <30 dB HL from 0.5 to 4 kHz). None of the participants reported acute neurological or psychiatric conditions. The study was conducted in agreement with the declaration of Helsinki and was approved by the local ethical committee of the University of Oldenburg. Each participant gave written informed consent prior to the experiment.

### Experimental design

The aim of the experiment was to elicit auditory evoked potentials (AEP) to analyse the N100 AEP. Participants therefore listened passively to auditory stimuli presented in a free-field setting. The experiment was conducted in a sound-shielded booth and participants were seated 1.5 m in front of a 24 inch monitor looking at a fixation cross. The auditory stimulus was a narrowband noise with a center-frequency of 1 kHz, a bandwidth of 100 Hz and a sampling frequency of 44.1 kHz. The narrowband noise had a duration of 400 ms and was presented through two high-quality speakers positioned at 45° azimuth in front of the subject. Prior to the experiment, intensity of the stimulus was adjusted individually to a comfortable loudness level in steps of 1 dB; loudness adjustment started at 79 dB(A). In total, 60 trials were presented with a jittered inter-stimulus-interval between 1,500 and 2,000 ms.

### EEG acquisition

Electroencephalography (EEG) data were collected from a 64 Ag/AgCl electrode cap with an equidistant sensor placement (Easycap, Herrsching, Germany) and a BrainAmp EEG amplifier system (BrainProducts, Gilching, Germany). In our experience, equidistant electrode placement based on infra-cerebral spatial sampling facilitates source localization efforts by a better coverage of the head sphere, although systematic comparisons to traditional 10–20 electrode layouts were not conducted (Hine and Debener, 2007; Debener et al., 2008; Hine et al., 2008; Hauthal et al., 2014; Stropahl and Debener, 2017). The nose-tip was used as reference and a central fronto-polar site as ground. To capture eye blinks and eye movements, two electrodes were placed below the eyes. Electrode impedances were kept below 20 kΩ. Sampling rate of EEG recording was 1000 Hz and online filters from 0.016 to 250 Hz were applied. Stimulus presentation was controlled with Presentation software (Neurobehavioral Systems, Albany, CA, USA).

### Analysis pipeline and data sharing

The EEG data of the 10 participants and the analysis scripts are available at https://figshare.com/s/48f8d9de715bafa5811b. The scripts and the detailed step-by-step tutorial are also available within the Supplementary Materials. Included is the EEGLAB code for the pre-processing of EEG data, including artifact attenuation using ICA (Bell and Sejnowski, 1995; Jung et al., 2000a,b) and CORRMAP (Viola et al., 2009). Furthermore, the Brainstorm source estimation pipeline was scripted and includes functionality for a group-level analysis. Note that a manual set-up of the Brainstorm database is necessary. A screenshot of the settings for the database used here can be seen in Supplementary Figure S2 (cf. Brainstorm tutorial on creating a new protocol http://neuroimage.usc.edu/brainstorm/Tutorials/CreateProtocol). To use the BEM head model, OpenMEEG has to be installed, which needs an active Internet connection (see Brainstorm Tutorial on head modeling http://neuroimage.usc.edu/brainstorm/Tutorials/HeadModel).

The use of the provided script requires that users have at least basic understanding of Matlab and signal processing, as well as of EEG analysis. The provided scripts are under the MIT license and are provided without warranty of any kind. We do not take any responsibility for the validity of the application or adaptation of this code, or parts thereof, on other datasets.

## Stepwise procedure

Please download the analysis scripts as well as the EEG raw data here https://figshare.com/s/48f8d9de715bafa5811b (.zip). After unzipping the archive including all necessary files, Matlab needs to be opened and the current Matlab folder should be changed to the/scripts directory.

### EEG data analysis

**Figure 1:**
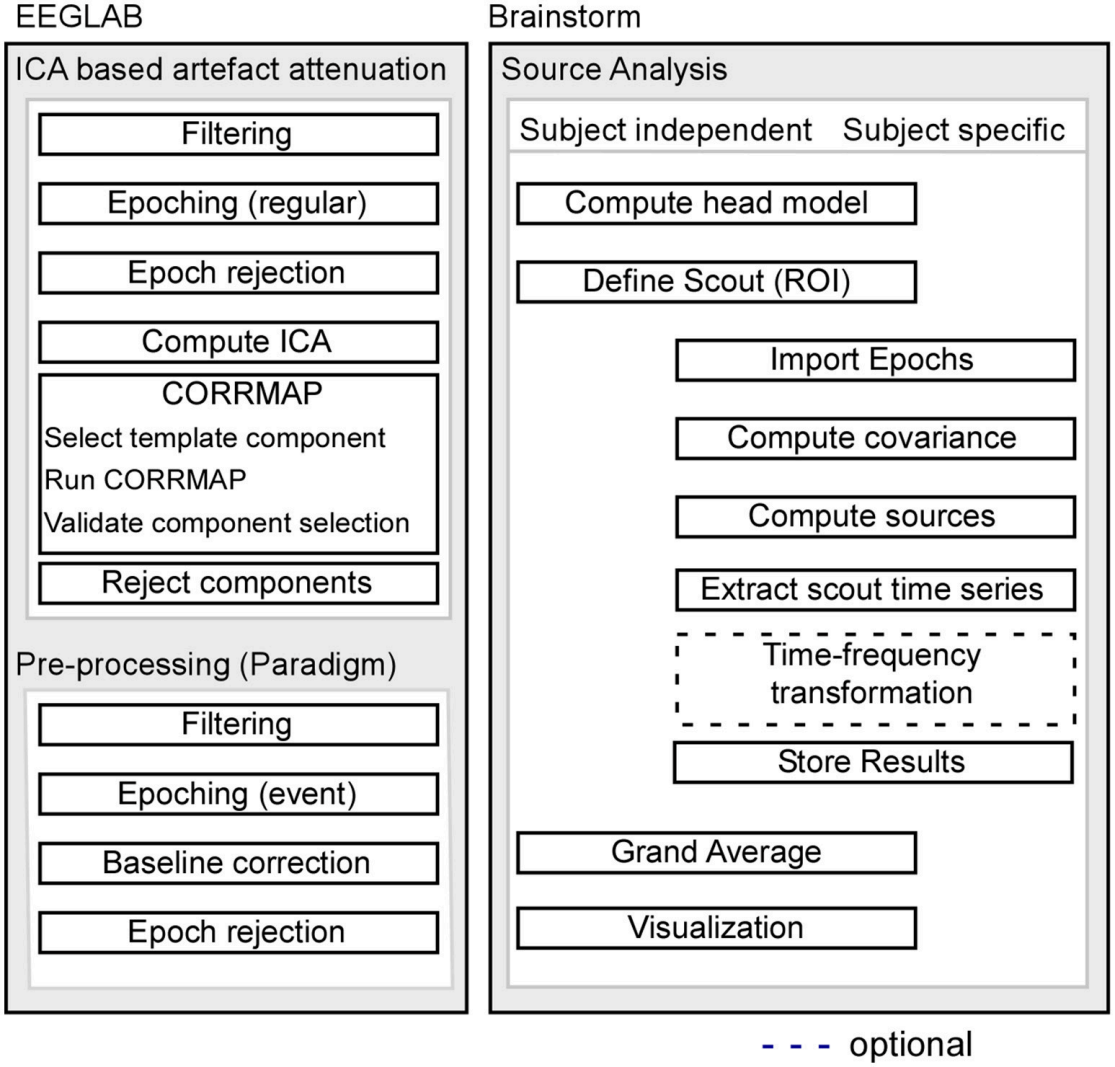
Schematic illustration of the processing pipeline. The dashed line indicates that alternative processing steps are possible, but are not implemented in the current pipeline. EEG pre-processing using EEGLAB is shown on the left, while source analysis implemented in Brainstorm is shown on the right.

Pre-processing of EEG data was performed using custom scripts and EEGLAB 13.6.5b (Delorme and Makeig, 2004) within the Matlab environment (Mathworks). For a schematic illustration of the processing pipeline, see Figure 1.

#### Step 1

The EEG raw data files (.vhdr, BrainAmp file format) are transformed to EEGLAB (.set) files. Execute the script ana00_convert_rawdata.m.

#### Step 2

Raw EEG data was subjected to an independent component analysis (ICA), based on the extended Infomax (Bell and Sejnowski, 1995; Jung et al., 2000a,b), to attenuate stereotypical artifacts such as eye blinks, lateral eye movements and electrical heartbeats (cf. script ana01_ICA.m).

ICA decomposition can be improved by high-pass filtering (Winkler et al., 2015) and by excluding data segments that contain rare and non-stereotypical events (Debener et al., 2010). In order to improve the ICA decomposition quality, data were low-pass filtered (windowed sinc FIR filter, cut-off frequency 40 Hz, filter order 500) and high-pass filtered (windowed sinc FIR filter, cut-off frequency 1 Hz, filter order 100; Widmann and Schröger, 2012; Widmann et al., 2014). To reduce computation time, data were then re-sampled to 250 Hz. In order to identify non-stereotypical events, continuous datasets were segmented into consecutive epochs with a length of 1 s. Epochs with a joint probability larger than three standard deviations (SD) were rejected prior to computing the ICA. The choice of this parameter was based on our lab standard. Please see Step 4 for further explanation of parameter choices for artifact correction. ICA was performed using the option ‘extended’, enabling the algorithm to extract sub-Gaussian and super-Gaussian sources (Lee et al., 1999). In our experience, this option can enhance the representation of noise sources and thereby improve artifact attenuation quality. The option ‘PCA’ reduced the number of components decomposed from 64 to 50. This step is not necessary but reduces computation time. Note that large datasets, and analyses strategies aiming for particular brain signals contributing little variance to the overall recordings, may benefit from decomposition without dimensionality reduction. The resulting ICA weights were then applied to the original, unfiltered, continuous data set, to allow for a paradigm-specific pre-processing (see below).

#### Step 3

Subsequently, ICA components representing artifacts were identified using the semi-automatic algorithm CORRMAP (Viola et al., 2009; cf. script ana02_corrmap.m). CORRMAP is compatible with EEGLAB and can be used as a plug-in (https://sccn.ucsd.edu/wiki/EEGLAB_Plugins). The algorithm clusters ICA components with a similar topography in all datasets based on a manually selected template component (see Supplementary Figure S3 for an exemplary CORRMAP output). Similarity between all ICA components and the user-selected template component is computed by a correlation of the ICA inverse weights (Viola et al., 2009). Here, the applied threshold criterion for selection was a correlation coefficient of *r* ≥ 0.8, which is the default value in CORRMAP and which has been proven to reveal accurate results. It is known that physiological processes such as eye blinks can be represented in more than one (but typically <4) ICA components, especially if high-density EEG recordings are used. CORRMAP therefore includes a parameter to define how many components could represent the same artifact (Viola et al., 2009). The maximum number of components that can be selected within one dataset was here set to three. Selected components were removed from each continuous EEG data sets (see Supplementary Figure S4). A more recently developed toolbox named Eye-Catch (Bigdely-Shamlo et al., 2013) seems to perform at par with CORRMAP and provides a fully automatic eye –component detection procedure.

#### Step 4

After cleaning the continuous data from stereotypical artifacts with ICA, EEG data sets were filtered with a low-pass (windowed sinc FIR filter, cut-off frequency 40 Hz, filter order 100) and a high-pass (windowed sinc FIR filter, cut-off frequency 0.1 Hz, filter order 500; cf. script ana03_preprocesing.m). Data were segmented relative to sound onset into 1s epochs (−200 pre-stimulus onset to 800 ms post-stimulus onset) and all epochs were corrected to a pre-stimulus baseline of 200 ms. Remaining artificial epochs not accounted for by ICA-based artifact attenuation were identified and rejected. We used the method of joint probability, which calculates the probability distribution of values regarding all epochs. Segments that contain artifacts are likely to show a difference in occurrence and can therefore be detected with this method. The parameters are set to a rather conservative threshold of 4 standard deviations to avoid selecting epochs containing useful data (joint probability of >4 SD; see Supplementary Table S1 for rejected epochs). The parameters were set according to our lab standards and the experimental conditions. Be aware that the choice of parameters depends on the quality of your EEG data, the experimental design and the analysis to be performed.

### Source analysis

#### Step 5

Cortical source activations were estimated using Brainstorm software (Tadel et al., 2011). Brainstorm uses a distributed dipoles model as fitting approach. For the current experiment, the method of dynamic statistical parametric mapping was applied to the data (dSPM, Dale et al., 2000). dSPM tends to localize deeper sources more accurately than standard minimum norm procedures, but the spatial resolution remains low (Lin et al., 2006). The dSPM method uses the minimum-norm inverse maps to estimate the locations of the scalp-recorded electrical activity and works well, in our experience, for modeling auditory cortex sources.

After EEGLAB based pre-processing (artifact attenuation, filtering and epoching, output from ana03_preprocessing.m), the EEG data were imported into Brainstorm (cf. script ana04_brainstorm.m Part 1). Note that in this pipeline no individual anatomies and no individual electrode locations are used. Instead one general electrode location file was used for all participants. For this, the exact positions of all cap electrodes were first digitized (Xensor electrode digitizer, ANT Neuro, The Netherlands) and the measured electrode locations were then visually inspected and manually corrected to fit the default anatomy using the Brainstorm graphical interface.

Single-trial pre-stimulus baseline intervals (−200 to 0 ms) were used to calculate single subject noise covariance matrices and thereby estimate individual noise standard deviations at each location (Hansen et al., 2010). The boundary element method (BEM) as implemented in OpenMEEG and was used as a head model using Brainstorms default parameters. The BEM model provides three realistic layers and representative anatomical information (Gramfort et al., 2010; Stenroos et al., 2014). For source estimation, the option of constrained dipole orientations was selected, which models one dipole, oriented perpendicular to the cortical surface for each vertex (Tadel et al., 2011). EEG data were re-referenced to the common average before source estimation, which is a default pre-processing step in most source analysis software. The main reason for re-referencing to the common average is to fulfill the assumption that a net source activity of zero current flow is achieved to not bias source strength estimates (cf. Michel et al., 2004). The single-trial EEG data is averaged for each participant and the estimate of active sources is performed on the subject average.

### Extracting source activity time series

For the definition of a region-of-interest (ROI), the Destrieux atlas (Destrieux et al., 2010) as implemented in FreeSurfer (http://ftp.nmr.mgh.harvard.edu/fswiki/CorticalParcellation) and available in Brainstorm was used, as no individual anatomies of the participants were available. An auditory ROI was selected, corresponding to the ‘*S_temporal_transverse*’ scout in the Destrieux atlas. Individual peak activation of the N100 AEP in the auditory ROI were extracted and analyzed on a group level for both the right and left hemisphere (cf. script ana04_brainstorm.m Part 2–3).

Brainstorm offers the possibility to use predefined scouts (atlas based), or to manually define a region of interest either anatomically or functionally (e.g., based on the activation pattern of a localiser task). To illustrate this option, a second ROI was defined based on the source level activity. For this, the source level average group activation was calculated in Brainstorm, and the region around the maximal activity on the auditory cortex was used as center of the ROI (scout). In the current approach, the scout was defined manually by visual inspection. However, there are several options to define a scout. For example a statistical test that differentiates the activation of the baseline and the N100 peak could be applied (using statistical functions in Brainstorm). This approach gives a more objective way of defining the activation region for the N100 component. A similar approach to define a scout can be applied for comparing conditions or groups of subjects. The atlas-based and the manually defined activity-based scouts for the left and right hemisphere, have here a similar size (between for 10 and 15 vertices), which allows better comparison.

### Time-frequency analysis

Brainstorm gives the option to perform a time-frequency analysis of the estimated source activation (cf. ana04_brainstorm_TF). The first steps are similar to the previously explained pipeline with the difference that time-frequency decomposition is computed on the single trial source estimates for each subject. The parameters for the time-frequency decomposition depend on the experimental design and the default settings in Brainstorm may be a good starting point (cf. Brainstorm tutorial on time-frequency analysis http://neuroimage.usc.edu/brainstorm/Tutorials/TimeFrequency). For the here presented pipeline, time-frequency decomposition is an optional processing step. Due to experimental constraints, no time-frequency results are shown in this pipeline. Please be aware, that the scripts ana04_brainstorm.m and the one with time-frequency decomposition (ana04_brainstorm_TF.m) should be used in separate brainstorm databases, otherwise the correct functionality could not be guaranteed.

### Visualization of results

#### Step 6

Calculation and visualization of sensor level group results (EEGLAB) is done based on the pre-processed data (output of ana03_preprocessing.m). ERP time courses and topographies for different latencies are plotted with the help of the EEGLAB gui.

#### Step 7

Calculation and visualization of source level group results (Brainstorm) is done based on the estimated source activity (output of ana04_brainstorm.m). The grand average source level activity is depicted as well as the grand average time series of the pre-defined regions of interest (scouts).

To reproduce the figures shown in this manuscript follow the steps explained in details in the Supplementary Materials. Additionally, a statistical comparison of the estimated time course of the left and the right scout was performed. Though, this is not the focus of this tutorial the intention was to briefly show this option in brainstorm using the gui (see Supplementary Material Step 7).

## Results

In the next section, the results will be presented following the previously explained analysis pipeline.

### ICA decomposition

**Figure 2:**
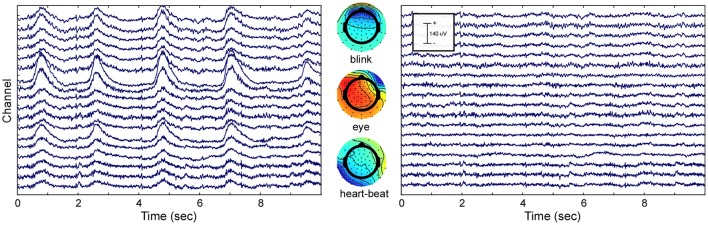
ICA based artifact attenuation. Left) Original EEG time course, shown for a subset of 18 electrodes and 10 s. Center) ICA topographies representing eye-blinks (Top), lateral eye movements (Middle) and heartbeat (Bottom). Right) EEG data after ICA based artifact attenuation. The EEG time courses were reconstructed excluding the identified artifact components.

To show the effect of ICA decomposition on the raw EEG data, one exemplary dataset is illustrated (see Figure 2). The graphic shows 10 s of multi-channel raw EEG data of a subset of 18 channels before (left part of Figure 2) and after removing selected ICA components (right part of Figure 2). Three ICA components representing eye-blinks, eye-movement and heartbeat were identified with the CORRMAP algorithm (middle part of Figure 2).

The components presented in Figure 2 represent common artifacts that can often be identified by ICA in raw EEG data despite different experimental and electrode set-ups (Debener et al., 2010). These components usually have distinct topographies as well as time courses, which simplifies their identification. Other components, such as event-related components or other less stereotypic artifactual components are often more difficult to distinguish. The removal of artefictual components by back-projection reveals a new, artifact-attenuated EEG data set (see Figure 2, right part), which is suitable for further processing. Note that we provide here a realistic example; ICA artifact correction may outperform other procedures but is not perfect. Hence, residual artifact may remain in the data (cf. Figure 2), or brain-features of interest may be lost during ICA correction. An additional comparison of pre-processed EEG data (grand average ERP of all data sets) with and without ICA artifact correction is shown in the Supplementary Materials.

### EEG analysis

**Figure 3:**
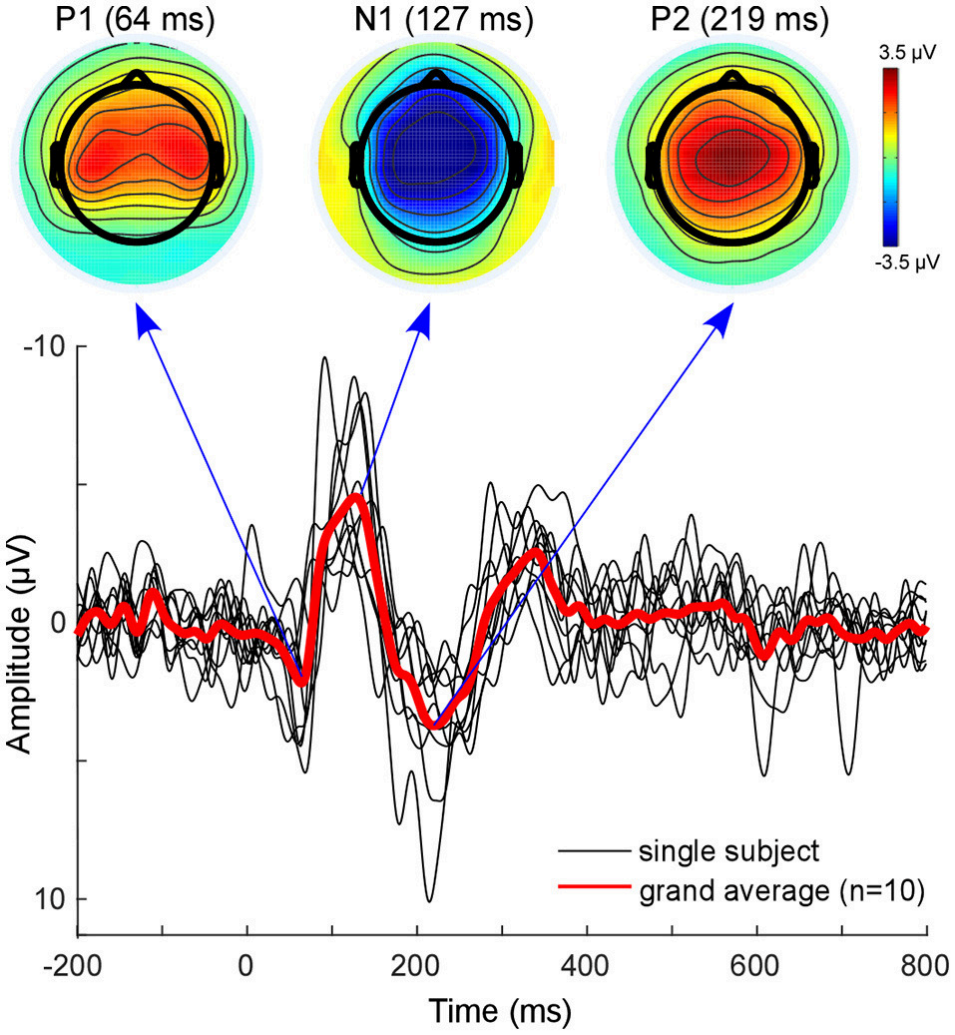
Sensor level analysis. Shown is the grand average (Red line) of all subjects as well as single subject AEPs. Additionally, the grand-average topographies for the P100 component and the N100-P200 complex are plotted on top. Figure is made with the Matlab function plot.m and the EEGLAB function topoplot.m.

The classical EEG sensor level analysis shows the expected auditory evoked response to the presented auditory stimulus (Figure 3). The morphology of the grand average auditory evoked potential (AEP) shows the characteristic components with prominent peaks at 64 ms (P100), 127 ms (N100), and at 219 ms (P200). The overall morphology is consistent across single subjects (Figure 3, black lines). The topographies obtained at peak latencies are also characteristic for the respective ERP components (Figure 3, top). The P100 is known to correspond to an early sensory response to the auditory stimulus, and is reflected as a positivity over central electrodes. The P1 is often used in specific paradigms to test suppression effects, e.g., in schizophrenic patients (Sur and Sinha, 2009).

The N100 is mostly distributed over more fronto-central electrodes, and is known to be mainly generated from primary auditory cortices (Näätänen and Picton, 1987; Zouridakis et al., 1998). The P200 component is reflected as a positive-voltage deflection prominent over the vertex electrode. A recent study provided evidence that N100 and P200 have distinct generators in the auditory cortex (Ross and Tremblay, 2009). The P200 seems to be generated in more anterior regions of the auditory cortices compared with the N100. This might be reflected in the activation shift observed in the topographies. Nevertheless, due to the inverse problem, sensor-based EEG data cannot reveal accurate spatial information with regard to which sources are involved (Lopes da Silva, 2013). EEG source localization is one tool aimed toward overcoming this problem. However, findings of adjacent and overlapping but partly different generator sites for N100 and P200 may be difficult to obtain from EEG and were mainly observed with MEG.

### Source localization

**Figure 4:**
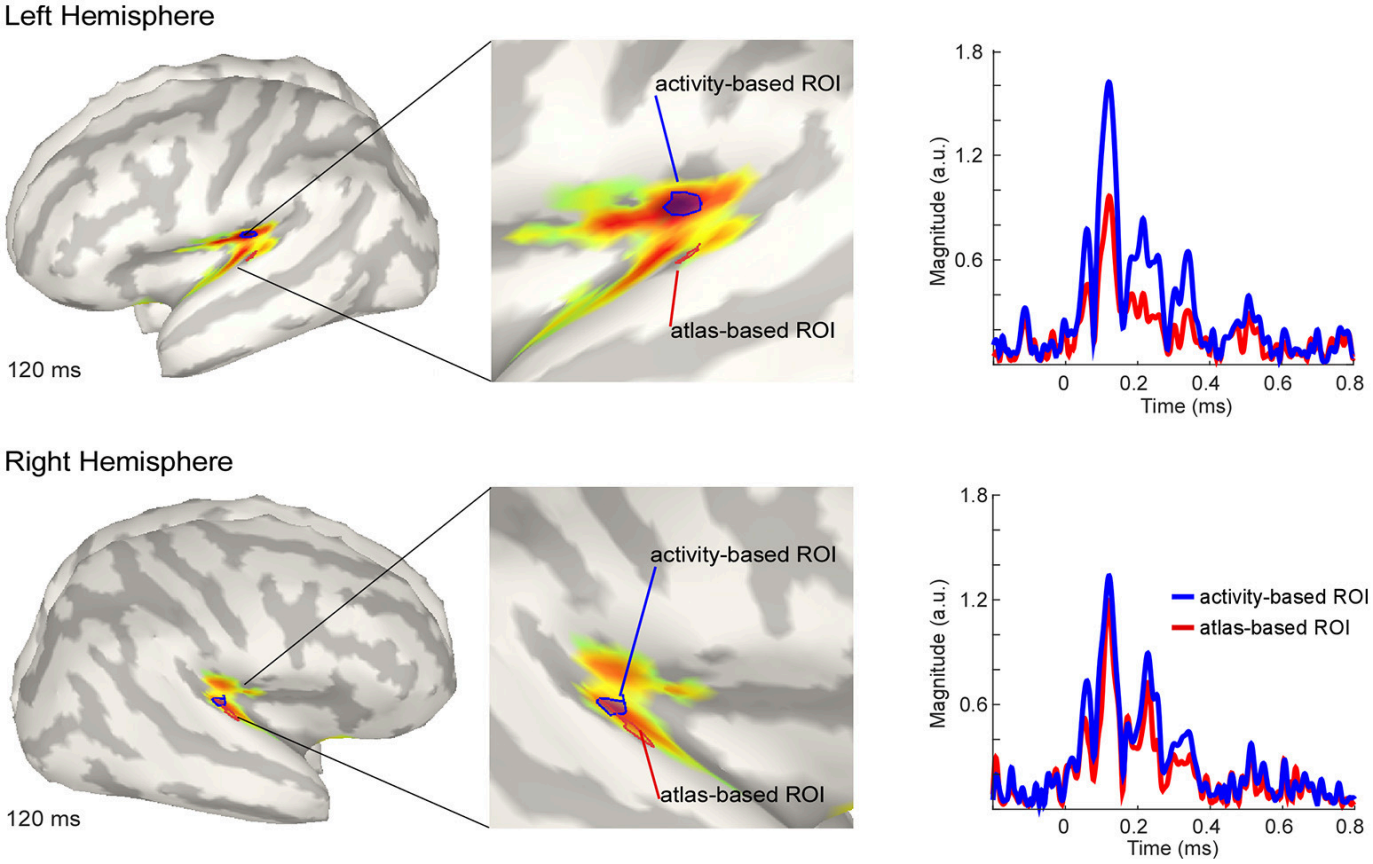
Grand average source level activity for the N100 component. Shown is the activation at the latency of the N100 peak for the left hemisphere (Top) and the right hemisphere (Bottom) for an activity-based ROI (Blue) and a (Destrieux) atlas-based ROI (Red). The middle part of the figure shows a zoomed view of the ROIs for a better visualization. Activation is shown as absolute values with arbitrary units based on the normalization within the dSPM algorithm. Right Colum: Time series of the activation in the atlas-based ROI (Blue) and the activity-based ROI (Blue). Activation is shown as absolute values and in arbitrary units, as provided by the normalization within the dSPM algorithm.

The estimated active sources of the EEG data are shown in Figure 4. Here the peak activation of the N100 of the right and the left hemisphere (top) for an atlas-based ROI (red) and an activity-based ROI (blue) is plotted. The data of cortical activation is shown as absolute values with arbitrary units based on the normalization within the dSPM algorithm. For the left hemisphere, the atlas-based ROI does not fully capture the hotspot of the source level activity. The activity-based ROI is located deeper, adjacent to, but outside of the auditory cortex, pointing toward EEG spatial resolution limitations. However, additional activation in the auditory areas can be readily observed, and the overall activity pattern is compatible with the interpretation of one, or several adjacent, sources in the auditory cortex.

The activation in the atlas-based ROI is, as expected, lower compared with the activity-based ROI (see Figure 4, right column). EEG source localization may have an accuracy of ~2 cm in ideal conditions (i.e., with a large number of electrodes and an individual head model Klamer et al., 2014). Due to missing individual anatomies, interpretation of the exact location of the activity-based ROI should be considered with caution. A similar but smaller pattern of magnitude difference between the atlas- and the activity-based ROI as in the left hemisphere was revealed for the right hemisphere. The activity-based ROI of the right hemisphere is located closer to the atlas-based ROI compared with the left hemisphere (Figure 4, lower part). Due to similar location of the ROIs the magnitude of activity does not differ as much as over the left hemisphere (Figure 4, right column).

## Discussion

The analysis pipeline presented here provides the option to process raw EEG recordings with ICA and additional pre-processing steps, to achieve good quality EEG data. The data can be further used to analyse effects on sensor space as well as to estimate the location of active neural sources.

The results obtained on the sensor and the source levels are in line with previous AEP work. AEP morphology and topographic maps of the sensor level data represent the AEPs as known from previous literature (Luck, 2005). Moreover, the AEP N100 is localized here to the supratemporal plane, which is in line with earlier reports for combined EEG/MEG data (Shahin et al., 2007; Gramfort et al., 2013). As generators of neural activity cannot unambiguously be interpreted from sensor EEG data, the transition from sensor to source space may facilitate interpretation of EEG results.

### The analysis pipeline

The pipeline we propose facilitates EEG source modeling by taking care of the consistent processing of all datasets and by implementing important EEG pre-processing steps. However, we do not claim that the pipeline outperforms other approaches, or is suitable for other paradigms and datasets. We claim, however, that the combination of two well-established Matlab toolboxes, each of them having their specific merits, can be advantageous. EEG pre-processing, including ICA based artifact attenuation, filtering and epoching as well as the sensor level analysis can be easily performed using EEGLAB. EEGLAB is a well-established and widely used EEG analysis toolbox that allows extensive signal processing in sensor and ICA component space. EEGLAB can be used either via a graphical user interface or the command line, and therefore allows easy access for novice users as well as extensive scripting capabilities for advanced users. EEGLAB has also a well-defined interface for implementing and sharing own extensions or plug-ins addressing specific signal processing challenges, such as CORRMAP (Viola et al., 2009) or CIAC (Viola et al., 2012), to name our own contributions.

Regarding EEG source level analysis we prefer using Brainstorm as it provides extensive source modeling capabilities and advanced, high-quality tools for visualization of source-modeled data. The combination of these two toolboxes provides an easy-to-work-with processing pipeline, specifically tailored for the purpose of traditional *sensor* space and subsequent, advanced *source* space analyses. With the detailed description and the scripts in the method section it should be fairly easy for the reader to reproduce the obtained results and to adapt the presented pipeline for their specific purpose.

The presented pipeline is flexible in its application. All parameters can be easily adapted to the specific research question. This includes, but is not limited to, the choice of the head model, the definition of scouts (region of interest) as well as subsequent signal analysis steps on source-level data. The source estimation can be computed either on the average over trials (as done here) or subjects, or on individual subject single trial data (as shown in script ana04_brainstorm_TF.m). Single trial source time courses can be subjected to any kind of signal processing, such as basic time domain analysis, time-frequency transformations or phase amplitude coupling.

### ICA artifact component identification

EEG data is typically contaminated with non-brain artifacts such as eye movement, heartbeat and muscle activity related artifacts. Some of these artifacts seem to continuously contribute to ongoing EEG signals (see, e.g., Fitzgibbon et al., 2015, for electromyogram contributions to the EEG). Consequently, a simple rejection approach, focusing on the removal of intervals with visible artifact, may not always suffice. Instead of rejecting data segments contaminated by stereotypical artifacts and thus losing a considerable amount of data, eye-related artifacts can be statistically modeled and subsequently removed from the data (Delorme et al., 2007; Viola et al., 2009). ICA separates the recorded data into multiple components, representing neural and non-neural sources. A good-quality decomposition allows identifying non-neural components with some experience. The data can be reconstructed without these components, which leads to an attenuation of unwanted sources. ICA artifact attenuation however requires distinguishing components that represent artifacts from components that contain signal of interest, a far from trivial problem. Here, we used CORRMAP, a semi-automated approach, which requires the manual selection of a single template component only. However, while fully automated identification of artifact components is possible (Bigdely-Shamlo et al., 2013) we recommend a careful visual inspection of the ICA decomposition and the resulting ICA based artifact attenuation.

### Source analysis

We used the ICBM152 anatomy to compute the head model, as no individual anatomies were available. Brainstorm also provides the possibility to use the MNI Colin-27 brain and the FSAverage by default, but every other anatomical model suited for the specific research question or population can be used. The lack of individual anatomical information is common for many EEG studies due to financial or time constrains, but EEG source modeling can be justified without individual anatomical information if the results are interpreted with care (Sandmann et al., 2012; Stropahl et al., 2015; Stropahl and Debener, 2017; Bauer et al., 2018). However, in general it seems beneficial to use individual anatomical information for EEG source modeling. With a large number of electrodes and an accurate head model the localisation accuracy of EEG can be comparable to MEG, but can be several centimeters otherwise (Klamer et al., 2014).

For the definition of the scouts, or anatomical regions of interest, we used the Destrieux surface based anatomical atlas, but other atlases are available as well in Brainstorm. Further, scouts can also be defined based on the activation itself (e.g., based on the activity of an independent localizer task), or by hand for a specific region of interest, either on group level or for each individual. Again, a word of caution is advised when using (pre-defined) scouts. Individual, or default, brain anatomy and functional localisations can differ, as shown in the present example. Specifically, predefined regions of interest may or may not match to a particular individual anatomy. The risk of mismatches between brain structure and estimated functional localization seems more prominent for small regions of interest, such as auditory cortex; for regions known to be characterized by large individual differences in anatomy, and thereby deviations from a default anatomy; for complex source configurations, such as source contributions from adjacent, but opposing patches of cortical sulci; and for regions where head model inaccuracies may be more likely to occur, such as near-by skull openings. As a result, we generally tend to interpret unexpected activation patterns such as insular cortex contribution to the AEPs (Figure 4) as reflecting limitations and possible errors of EEG source modeling, and interpret only expected activation patterns confirming a priori predictions (such as auditory cortex contributions to AEP N100).

## Conclusion

The aim of this paper was to provide a pre-processing and analysis pipeline for processing raw EEG data, starting from pre-processing to obtain cleaned and high-quality data up to advanced source modeling. While the pre-processing of the EEG data was implemented using the Matlab analysis toolbox EEGLAB, the estimation of source activity was performed with Brainstorm. The current analysis pipeline is neither dependent on individual anatomies nor on individual electrode positions and can be used for single subject or group level analysis. Moreover, the current pipeline is flexible and can be easily adjusted to the specific purpose of various experiments.

## Author contributions

Data acquisition and analysis was primarily performed by MS, SD, A-KB, and MB contributed to the analysis and interpretation of the data and the drafting of the manuscript.

### Conflict of interest statement

The authors declare that the research was conducted in the absence of any commercial or financial relationships that could be construed as a potential conflict of interest.

## Supplementary material

Commented MATLAB code for all reported analyses is available in the Supplementary Materials along with screenshots of necessary Brainstorm or EEGLAB settings.
