# Supplementary material for: Source-Modeling Auditory Processes of EEG Data Using EEGLAB and Brainstorm
Source: Front Neurosci. 2018 May 8;12:309. doi: 10.3389/fnins.2018.00309 (PMC5952032; doi:10.3389/fnins.2018.00309)
Supplement: Supplementary file 1 [file Data_Sheet_1.PDF]

Supplementary Material

Source-modelling auditory processes of EEG data using EEGLAB and Brainstorm

Maren Stropahl, Anna-Katharina R. Bauer, Stefan Debener, Martin G. Bleichner*

*** Correspondence:** Corresponding Author: martin.bleichner@uni-oldenburg.de

1 Resources

The following MATLAB code presents the processing pipeline from continuous raw EEG data, using EEGLAB, to estimate source activation of a pre-defined region of interest, using Brainstorm. The scripts are designed for the available data sets and allow reproducing the analyses reported in the corresponding article. The necessary software, EEG data and the scripts are available for download here: https://figshare.com/s/48f8d9de715bafa5811b

This tutorial assumes that you

- *have downloaded the zip file from figshare,*
- *use the Brainstorm and EEGlab versions that are included (**Software** directory). Otherwise make sure that you have downloaded all necessary toolboxes. In that case the EEGLAB plugins need to be downloaded manually (see below),*
- *use the given directory structure for reading and writing (see figure 1).*

*To make sure that all paths in each script are set correctly all scripts need to be started from the /**scripts** directory (the provided scripts will automatically return the path to the /scripts folder after completing the computation).*

Software/hardware dependencies include:

- MATLAB (scripts tested on version 2015a and 2016a)
- EEGLAB: eeglab14_1_1b
  - Biosig 3.3.0 plugin (downloaded from https://sccn.ucsd.edu/wiki/EEGLAB_Extensions)
  - corrmap 1.03 plugin (downloaded from http://www.debener.de/corrmap/)
  - If you use another version of EEGLAB or if the EEGLAB software is stored in another location on your computer, please make sure to change the corresponding lines in each script.
- Brainstorm v. 3.4 (20-Nov-2017)
  - Be aware that the Brainstorm developers constantly update the Brainstorm software. Basic functionalities should not be affected by any update but users could take advantages of ongoing improvements. However we suggest that for running the present tutorial to use the Brainstorm version that we uploaded on the file server.
- Windows PC (scripts may also work on different operating system, but have not been fully tested).

2 Analysis Overview

The protocol will guide you from the import of the raw data to the visualization of the group results on sensor and source level. Additionally, a brief statistical comparison of the right and left auditory scout is explained.

STEP 1: Conversion from BrainAmp data files (.vhdr) to EEGLAB .set files

STEP 2: Independent component analysis for artefact attenuation

STEP 3: Selection and removal of stereotypical artefacts reflected in ICA components using the semi-automatic CORRMAP algorithm

STEP 4: Pre-processing of EEG data (filtering and epoching)

STEP 5: Estimation of the source activation of the auditory cortex using brainstorm

STEP 6: Calculation and Visualization of sensor level group results (using the EEGLAB GUI) to reproduce figure 3 of the manuscript.

STEP 7: Calculation and Visualization of source level group results (using the Brainstorm GUI) to reproduce figure 4 of the manuscript. A statistical comparison between the time course of the left and the right auditory scout is performed.

3 Step-by-Step protocol

- Download all necessary software, the EEG rawdata and the analysis scripts from https://figshare.com/s/48f8d9de715bafa5811b (.zip)

- Unzip the archive

- Open Matlab and

- Change the current Matlab folder to the /**scripts** directory (the provided scripts will automatically return the path to the /scripts folder after completing the computation).

- <u>**STEP 1**: Conversion from BrainAmp data files (.vhdr) to EEGLAB .set files</u>
  - Open the script ana00_convert_rawdata.m and execute the entire code.
  - The script loads the raw EEG files (.eeg; .vhdr; .vmrk) from the 'rawdata_vhdr' folder and converts them into set files saved in the newly created 'rawdata' folder.
  - You will now find for each of the 10 datasets a .set and a .fdt file in the 'rawdata' folder.

- <u>**STEP 2:** Independent component analysis for artefact attenuation</u>
  - Open the script ana01_ICA.m and execute the entire code (depending on your computer this step can take quite some time)
  - The script will load the data from the 'rawdata' folder and perform the ICA. You will now find for each of the 10 datasets an _ica.set and an _ica.fdt file in the 'ana01' folder. Please note, a window pops up as soon as the ICA process starts with the option to interrupt the ICA process. Please do not press the button if you do not want to cancel the process.

- <u>**STEP 3:** Selection and removal of stereotypical artefacts reflected in ICA components using the semi-automatic CORRMAP algorithm</u>
  - Open the script ana02_corrmap.m
    - Execute part 1 and part 2 of the script
    - The script will load in the data from the **/ana01** folder and generate the visualization of the ICA components
    - In the directory /**data/ana01** you will find a .png file for each participant showing the topographic visualization of all ICA components. These plots are used to manually select the artefact templates
    - In this tutorial we use dataset s08 to select the template topographies for eye-blinks, heartbeats and lateral eye movements. For this step open /**data/ana02/s08_ica.png**. You see the topographies of the 50 ICA components for subject 08, similar to what you see in figure S2**. Based on figure S2 one would select component 1 as template for eye-blinks, component 10 as template for an ECG (heartbeat) artefact and component 17 as template for lateral eye movement. Please identify these corresponding topographies in your s08_ica.png result and note the component numbers.** Be aware that your components might look slightly different compared to the ICA results in this tutorial. Differences might occur in the polarity of the components (i.e., a reversal of the colours red and blue). Additionally the order of the components might change slightly. This is due to the runica algorithm, which always starts with a

random weight matrix. Moreover the data is randomly shuffled for each training step. However, the topographies components as well as their order should not be completely different between two ICA decompositions on the same data set. See figure S4 for an overview of the diversity of eye-blink, lateral eye movement and heartbeat components between participants. Please indicate the numbers of the identified components in lines 108-113 (ana02_corrmap.m) the dataset number and the ICA component number you have identified as templates.

- Execute part 3 and part 4 of the script ana02_corrmap.m
- A sample output of the Corrmap algorithms is shown in figure S3 and an overview of the components selected for rejection of all participants is shown in figure S4
- The results of this analysis step can be found in the folder **/ana02**. A _badcomps.png file, created for each participant, shows again the 50 topographies. At the bottom of each the plot the numbers of the components that are rejected is indicated. For each of the 10 datasets you find a _ica_cleaned.set and a _ica_cleaned.fdt file, which contain the artefact-attenuated datasets.

- **STEP 4:** Pre-processing of EEG data
  - Open the script ana03_preprocessing.m and execute the entire code
  - The script loads in the data from **/ana02**, performs the pre-processing including epoching and saves the pre-processed and epoched data in the folder **/ana03.** For each of the 10 datasets you find a _ep_ar.set and a _ep_ar.fdt file. You will find the info.mat file, which contains the variable *comps,* which stores the index of the ICA components per subject that were rejected, and the variable *rej_ep,* which stores the index of the epochs that were excluded for each subject. Further, you will find the sensor_level_ERP.study in this folder, which will be used in STEP 6 for the sensor level analysis using EEGLAB.

- **STEP 5:** Estimating the source activation of the auditory cortex using brainstorm
  - For this tutorial we assume that you do not have a brainstorm_db, all steps necessary for our analysis are described hereafter (a more detailed tutorial of setting up Brainstorm can be found here: http://neuroimage.usc.edu/brainstorm/Tutorials/CreateProtocol). If you already have your own brainstorm database, please make sure that you change and adapt the path names for the location of your database correctly (see ana04_brainstorm.m line 50-53).
  - Change the current Matlab folder to the /**Software/brainstrom3** directory
  - Start Brainstorm from the Matlab command line by typing: *brainstorm*
  - Select the brainstorm_db folder as your database directory
  - Create a brainstorm database from the Brainstorm GUI
    - Menu File > New Protocol (see supplementary figure S6)
    - Protocol name: *EEG_AEPs*
    - Default anatomy: *Yes, use protocol's default anatomy*
    - Default channel file: *Yes, use one channel file per subject (one run per script)*
  - Change the current Matlab folder to the /**scripts** directory (Do NOT close the brainstorm GUI)
  - Open the script ana04_brainstorm.m and execute entire script (if you are using brainstorm for the first time, this step will take some time due to downloading and processing of the OpenMEEG model). Alternatively you can open and execute the script ana04_brainstorm_TF.m to estimate the sources for each individual trial and to compute

the time-frequency decomposition of single-trial source estimates for the selected scouts (here auditory and occipital scouts). Make sure that you are using a new protocol if you want to run the script ana04_brainstorm_TF. Otherwise your output will be corrupted.
    - In the brainstorm GUI (Functional Data View) you now find the 10 datasets. Each dataset contains the single trial data, the individual source estimate, and the time course of the two regions of interest.

With the completion of STEP 5 all analysis steps of the proposed pipeline are done. The data is now ready for subsequent sensor and source level analysis. The two following steps (STEP 6 and STEP 7) will show you how to visualize the sensor level results (using EEGLAB, STEP 6) and the source level results (using Brainstorm, STEP 7). Moreover in STEP 7 a simple statistical comparison between the left and the right auditory scout is shown.

- **STEP 6:** Calculation and visualisation of sensor level group results (using EEGlab gui) to reproduce the results as shown in figure 3 of the manuscript.
    - Open EEGLAB from the Matlab command line type: *eeglab*
    - To load the pre-processed sensor level data (output of ana03_preprocessing.m)
        - Menu File > load existing study
            - **/data/ana03/sensor_level_ERP.study**
    - Study Menu > Precompute channel measures
        - From the 'List of measures to precompute' select ERPs
        - For the Baseline; type: *-200 0*
        - Press OK
    - To plot ERP time course: Study Menu > Plot channel measures (see figure S7)
        - Select channel to plot: All E01 (vertex electrode, corresponds to Cz)
        - Select subject(s) to plot: All Subjects
        - Press on 'Plot ERP(s)' (on the right side of the GUI)
        - Press OK
    - To plot the topographies: Study Menu > Plot channel measures (see figure S7)
        - Select channel to plot: Press 'Sel. all'
        - Select subject(s) to plot: All subjects
        - Press 'Params'
            - Multiple channels selection:
                - Select plot topography at time (ms); type: *64*
                - Press OK
        - Press on 'Plot ERP(s)' (on the right side)
        - Press OK
    - Repeat the above steps to create the topographies at 127 ms, and 219 ms
    - The results of both steps can be seen in figure S8. Note that the y-axis of figure 3 in the manuscript is inverted (negative is up) while for the EEGLAB output (figure S8 left) negative is down. Important for figure S8 (right): the colour scale in the resulting topography plot (S8 right) is different than in figure 3 of the manuscript. For figure 3 all topographies have the same colour scaling. The plots generated above are scaled to their respective minimum and maximum. Consequently, the colouring of the plot will be slightly different.

- **STEP 7:** Calculation and visualization of source level group results (using the brainstorm GUI) to reproduce the results as shown in figure 4 of the manuscript. Additionally, the grandaverage

source activation is computed as well as the time course of activation compared between the left and the right auditory scout.

- Visualize grand average source level activity
  - In the ‘Functional data’ view select all 10 datasets and drag them to ‘Files to process’
  - Select ‘Process Sources’
  - Click on ‘RUN’
  - From the ‘Add Process’ Dropdown menu select Average>Average files
    - Group files: *Everything*
    - Function: Arithmetic average: mean(x)
  - From the ‘AddProcess’ dropdown menu select Sources > Spatial Smoothing. Default parameters set by the software should be used for this process (“Use absolute values of source activations or the norm of the three orientations for unconstrained maps”; FWHM: 3mm). While other smoothing options are possible, this will change the corresponding file name.
  - Press ‘Run’
  - In the ‘Functional data’ view the Folder ‘Group analysis’ appears
  - Click on the + to expand the Folder
  - In ‘Intra-subject’ double click on ‘Avg:Avg: s01_ep_ar (10)|abs|smooth3 to show the group average source level activity. You can rotate the brain to show the right hemisphere by pressing ‘3’ on the keyboard.
  - See figure 8 (left) for the settings and the resulting visualization of the grand average source level activity visualization on the inflated right hemisphere (right)
    - *120* ms
    - *Surface options* is set to *Transp: 0%* and *Smooth: 100%.*
    - Data options are set to *Amplitude 53%* and a *Min size of 20*
  - It might be useful to create a grand-average file of the time-domain averages for each subject to easily browse to the important peaks of the segment.
    - In the ‘Functional data’ view select all 10 datasets and drag them to ‘Files to process’
    - Select ‘Process Recordings’
    - Select “avg” as filter
    - From the ‘Add Process’ Dropdown menu select Average>Average files
    - Group files: *Everything*
    - Function: Arithmetic average: mean(x)
    - Click on ‘RUN’

- Visualize grand average time series of scouts
  - Change to the ‘Scout’ tab on the right part of the Brainstorm GUI
  - From the dropdown menu ‘User scouts’ select Destrieux
  - Scroll to the end of the list and select the last two entries ‘LT S_temporal_transversal’ and ‘LT S_temporal_transversal’ (see figure 9 left)
  - Right-click on Avg:Avg: s01_ep_ar(10)|abs|smooth3 (In ‘Group analysis’ > ‘Intra-subject’)
  - Select ‘Cortical activations’ > ‘Scouts time series’
  - The resulting scout time series is shown in figure 9 (right)

- Compute statistical comparison between the left and right auditory scout

- Brainstorm also provides the possibility to perform statistics on the results. Though, this is not the focus of this tutorial we show briefly how a simple comparison between the time course of the left and the right scout can be performed in brainstorm using the gui.
- Change to the ‘Process 2’ tab (lower right side of the gui)
- For each participant drag the file ‘Avg: s*_ep_ar(55) | scouts (S_temporal_transverse R)’ into the right window at the bottom of the GUI, ‘Files B: Matrix [2]’
- For each participants draw the file ‘Avg: s*_ep_ar(55) | scouts (S_temporal_transverse L)’ into the left window at the bottom of the GUI, ‘Files A: Matrix [2]’
- Make sure that the order of the participants is the same in the left and right window, this is necessary for the statistical comparison (paired)
- Click on ‘RUN’
- From the ‘Add Process’ Dropdown menu select Test>Permutation test: Paired
- Set the ‘Time window’: 60.0 and 160
- Press ‘Run’
- In the GUI you find the result of the permutation test under ‘Intra-Subject’
- In ‘Intra-subject’ double click on ‘Perm t-test paired [60 ms, 160 ms]: 10 files vs 10 files (10). For this comparison there are no significant differences between the AEP of the left hemispheric and the right hemispheric scout.

4 Supplementary Figures and Tables

4.1 Supplementary Figures

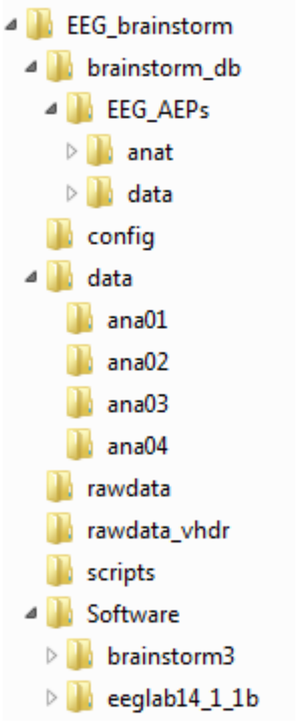

Supplementary Figure S1 Folder structure after all steps described in the step-by-step protocol are done. The directories ana01-ana04 and rawdata are created during the analysis and are not part of the download.

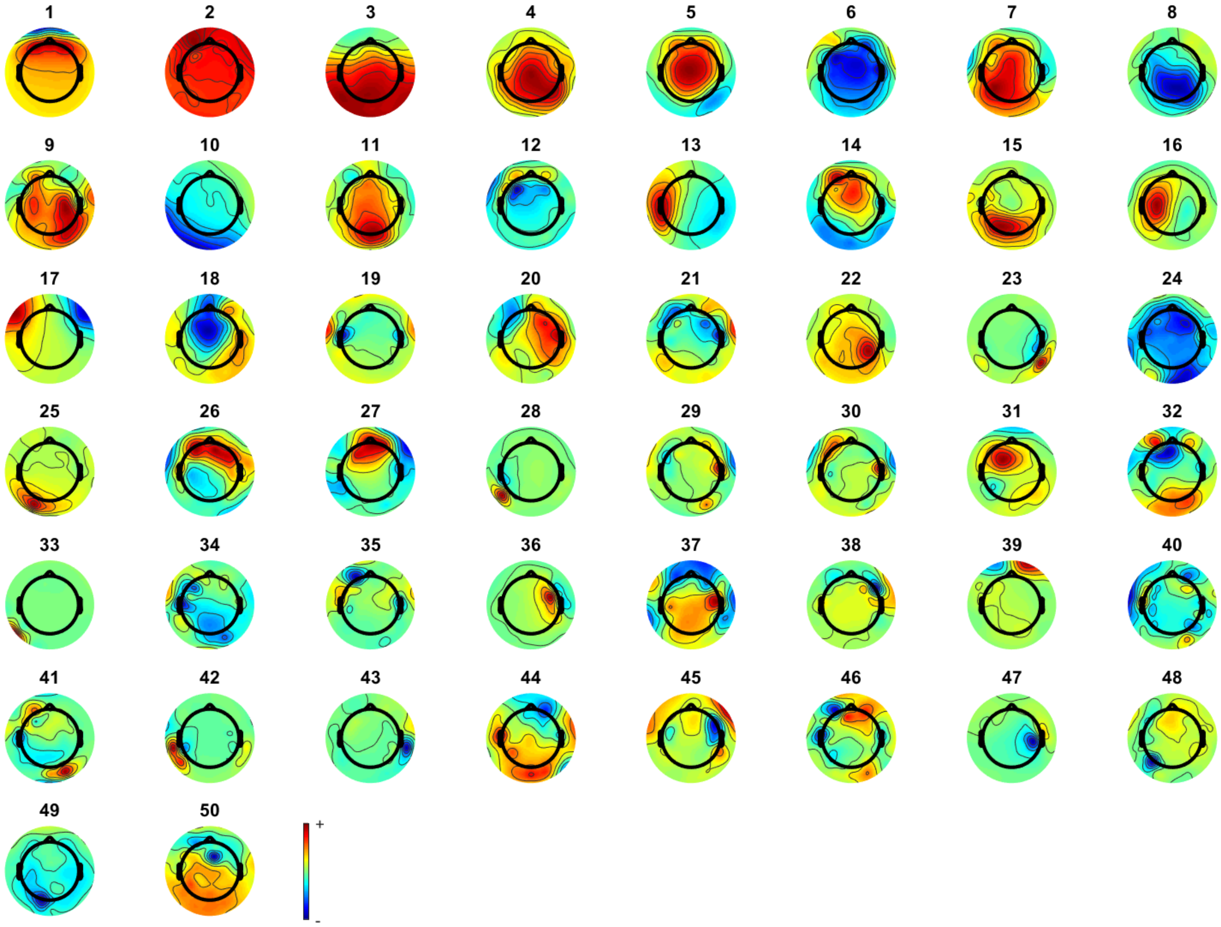

Supplementary Figure S2. Topographic representation of the ICA components for dataset s08. For the semi-automatic identification template topographies need to be selected. In this ICA run, component 1 is used as template for an eye-blink component, component 10 is used as template for a heart-beat component, and component 17 is used as a template for lateral eye movements. Important: when re-running the ICA decomposition the order as well as the polarity of the components might potentially change.

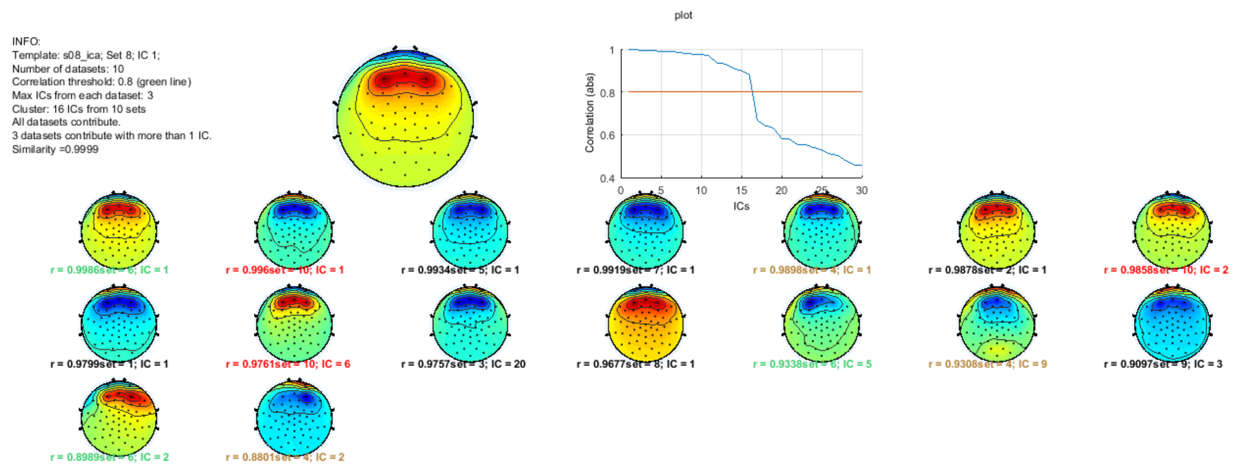

Supplementary Figure S3. Exemplary CORRMAP output for the semi-automatic identification of eye blink ICA components in the EEG data after running ICA. The selected template component is shown on top of the panel. The correlation threshold is set to 0.8. All data sets contribute with at least one component.

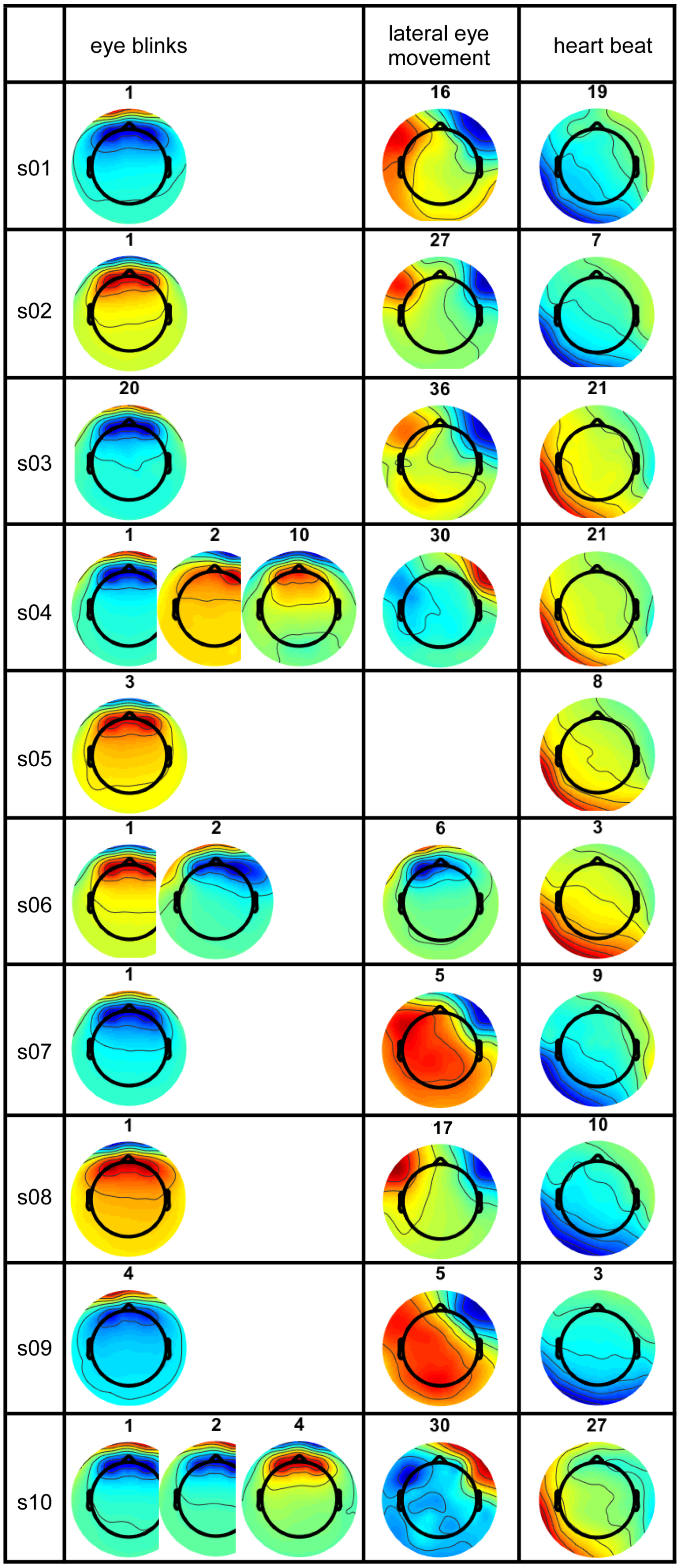

Supplementary Figure S4. ICA components selected for rejection by CORRMAP for each dataset. For some templates multiple components are found for some dataset. For dataset s05 no component for lateral eye-movements was detected. Mark that the numbering of components can change when you re-run the ICA analysis.

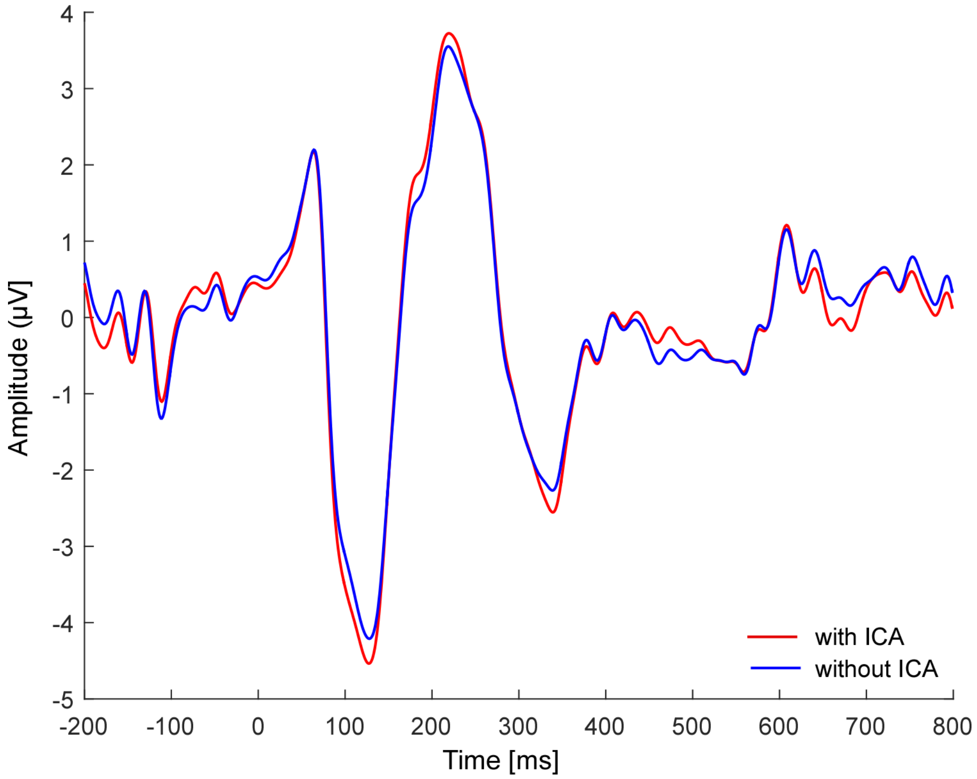

Supplementary Figure S5. Comparison of the grand average ERP (N=10 datasets) for the data pre-processed with ICA artefact correction (red line) and without ICA correction (blue line). Typically, the peaks of the ERP are enhanced (as the N1 here) and the noise of the baseline is reduced. The morphology however does not change.

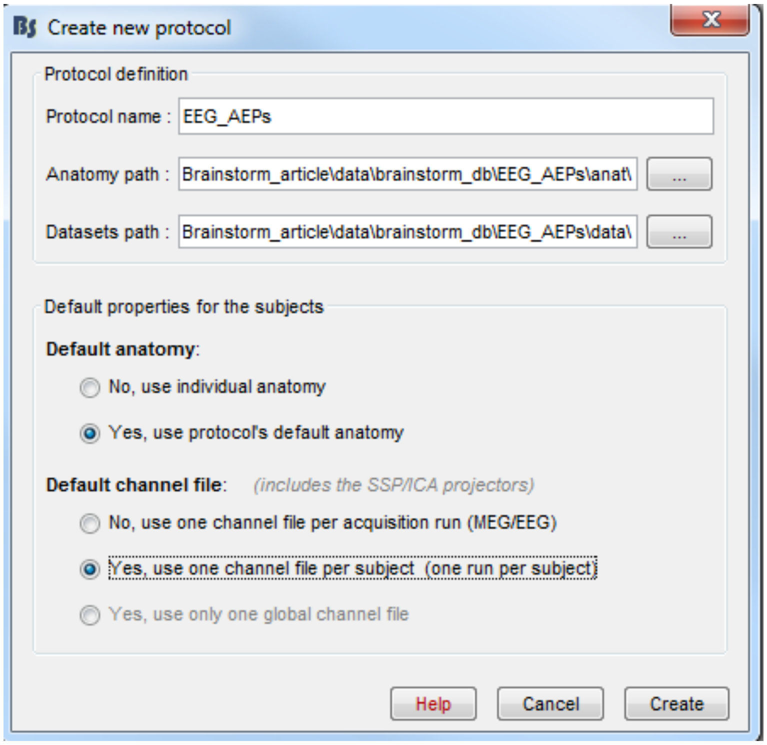

Supplementary Figure S6. Settings for the brainstorm database (cf. ana04_brainstorm.m). Parameters are adapted to the here presented EEG data without individual anatomies. Refer to tutorial 1 (http://neuroimage.usc.edu/brainstorm/Tutorials/) for further guidance on installing brainstorm and setting up a database.

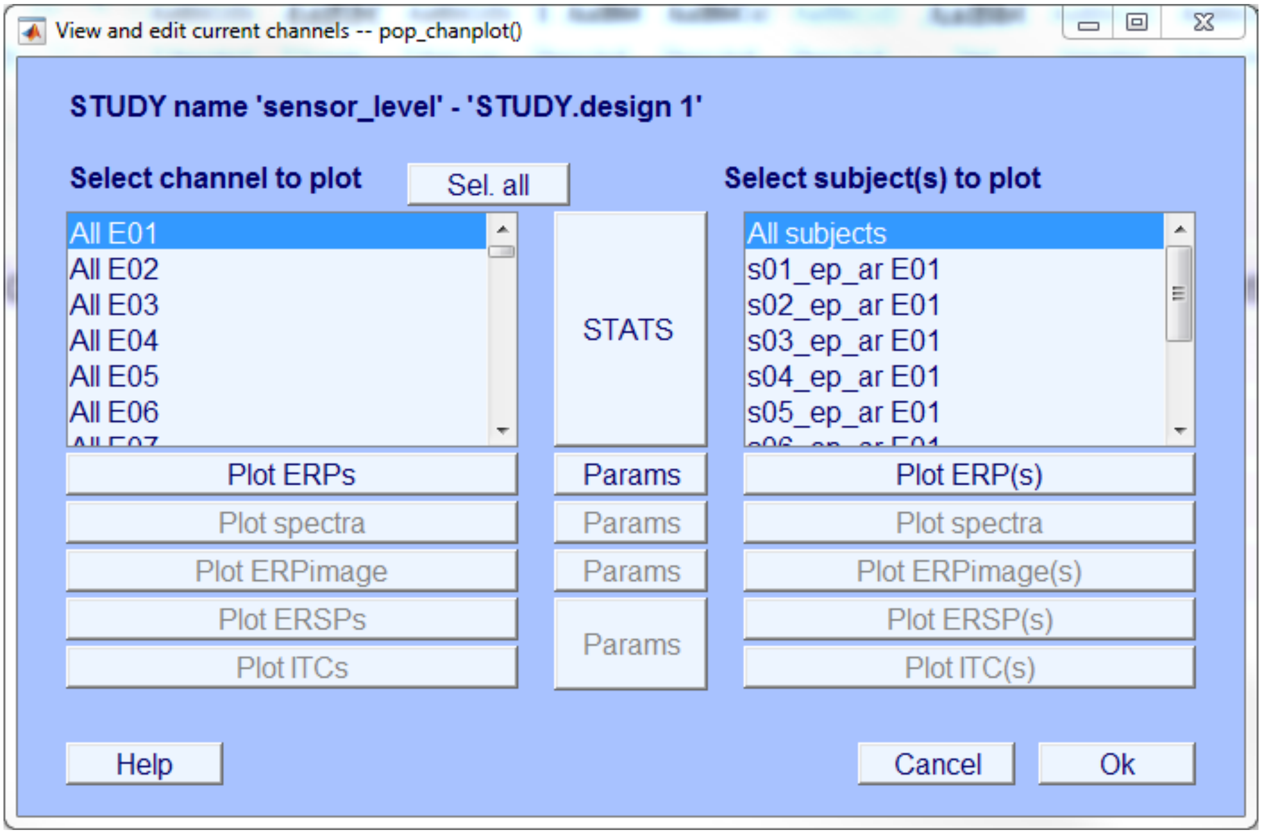

Supplementary Figure S7. EEGLAB GUI for visualizing group level results on sensor level.

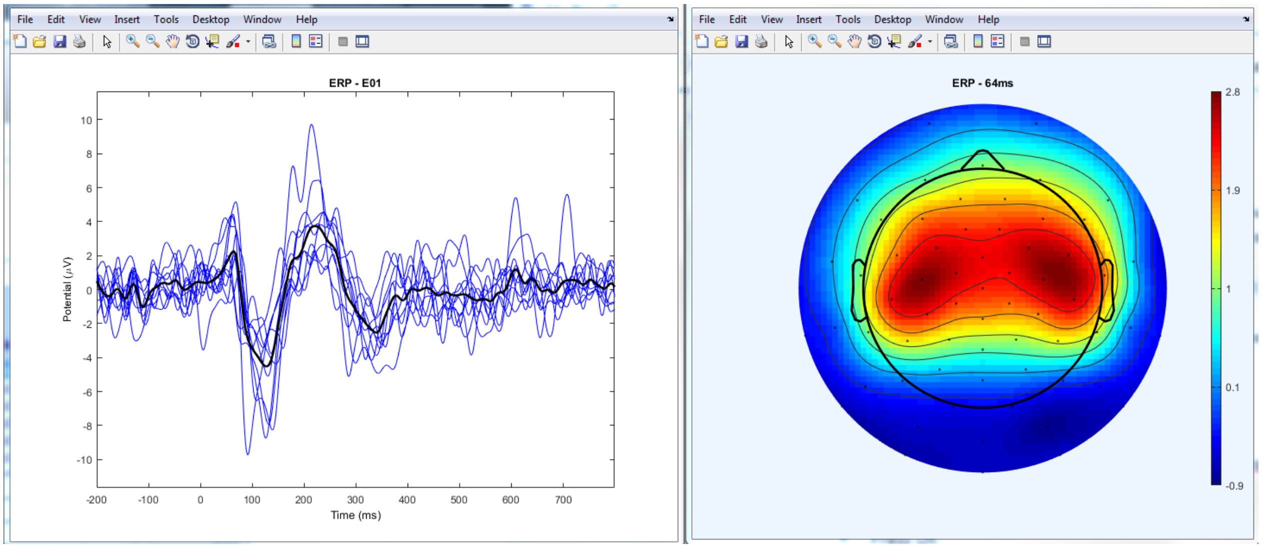

Supplementary Figure S8. Resulting visualization of the EEGLAB sensor level analysis. Left: The grand average time course (black line) of all subjects as well as single subject AEPs (blue lines). Right: The grand-average topography at 64 ms.

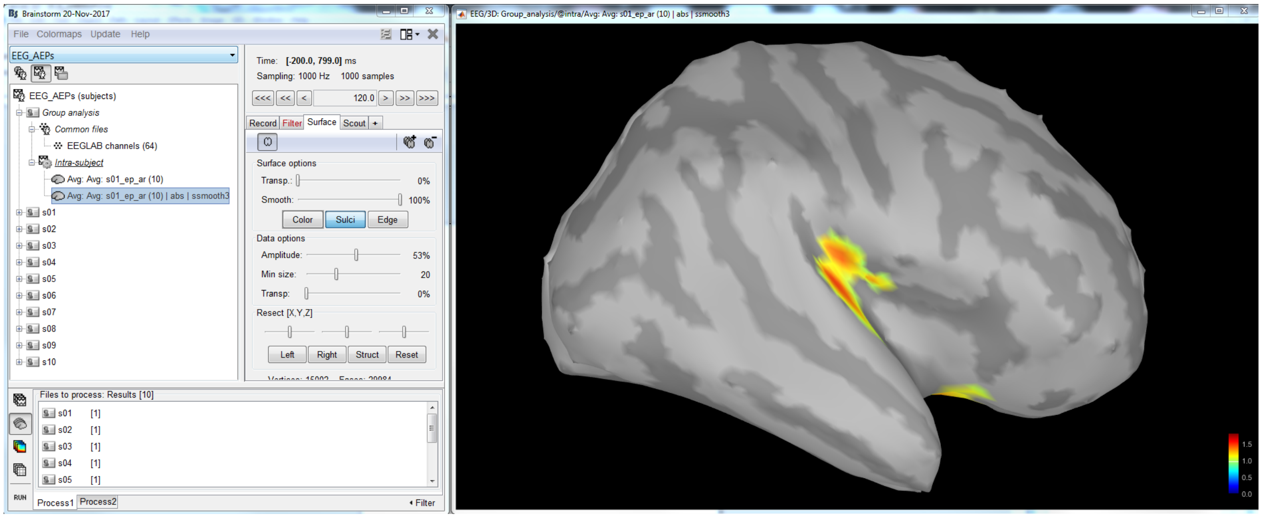

Supplementary Figure S9. Left: Brainstorm GUI state at the end of the first part of STEP 7 for visualizing the source level activity. The source level activity on the right is shown at *120* ms, *Surface options* is set to *Transp: 0%* and *Smooth: 100%*. Data options are set to *Amplitude 53%* and a *Min size of 20*. Right: Resulting visualization of the grand average source level activity on the inflated right hemisphere.

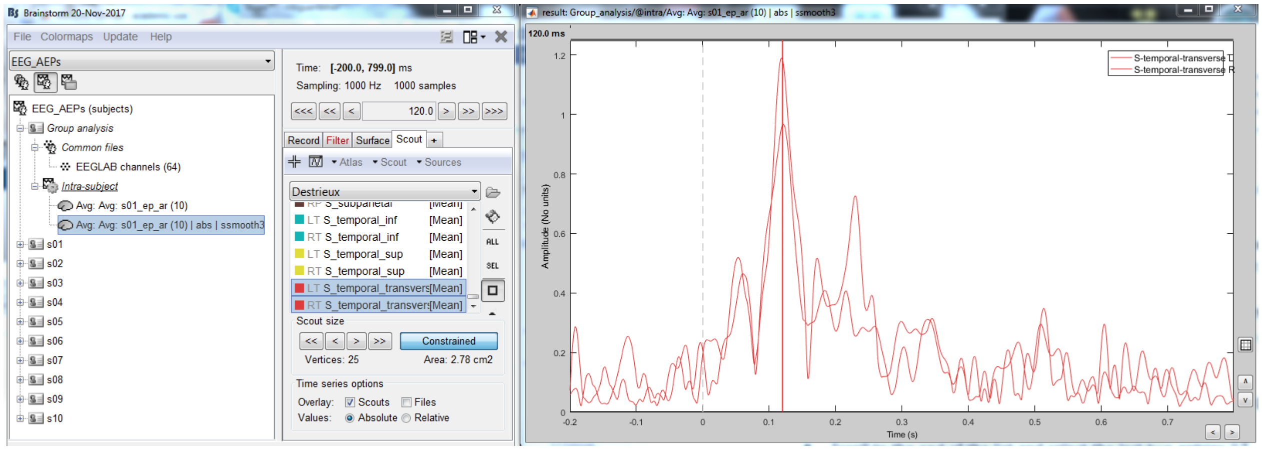

Supplementary Figure S10. Left: Brainstorm GUI state at the end of the second part of STEP 7 for visualizing the scout time series. Right: Resulting visualization of time series of the activation in the atlas-based ROI.

Supplementary Tables

Supplementary Table S1. Second column: Artefactual epochs that were rejected based on a joint probability criterion for each participant. See script ana03_preprocessing.m for the rejection of artificial data segments.

**Subject**	**Epoch (index)**
s01	38; 53
s02	36; 38
s03	6; 29; 40; 58; 59
s04	24; 42; 54
s05	20; 45; 59; 60
s06	1; 53; 56
s07	3
s08	1; 2
s09	8; 10; 14; 52; 54; 56; 59
s10	45; 54

5 Matlab scripts

5.1 Converting recording files to EEGLAB format: ana00_convert_rawdata.m

```
%%%%%%%%%%%%%%%%%%%%%%%%%%%%%%%%%%%%%%%%%%%%%%%%%%%%%%%%%%%%%%%%%%%%%%%%%%%%%%%
%%
% ana00_convert_rawdata.m
% Script 0:
%     loads in BrainAmp acquisition files and converts them to EEGALB set
%     files

% Output: EEG set-files
%
% Martin Bleichner 11/01/2018
%%%%%%%%%%%%%%%%%%%%%%%%%%%%%%%%%%%%%%%%%%%%%%%%%%%%%%%%%%%%%%%%%%%%%%%%%%%%%%%
%%

%% DIRECTORIES
clear; close all;
% define project folder

% execute this script from within the 'scripts' folder.
MAINPATH = fileparts(pwd);
PATHIN  = fullfile(MAINPATH,'rawdata_vhdr', filesep); % path containing
rawdata
PATHOUT = fullfile(MAINPATH, 'rawdata', filesep);    % path for script output

if ~exist(PATHOUT)
    mkdir(PATHOUT);
end
addpath(fullfile(MAINPATH,'Software','eeglab14_1_1b'))
chan_file = fullfile(MAINPATH,'config','elec_64ch.elp')

% Datasets
% Input files
cd(PATHIN)
list=dir('*.vhdr'); %reads all .vhdr files in that path
len=length(list); %total number of datasets that will be evaluated
subj={'s01','s02','s03','s04','s05','s06','s07','s08','s09','s10'};
[ALLEEG EEG CURRENTSET ALLCOM] = eeglab;
% Loop over subjects
for s=1:len

    name{s} = strrep(list(s).name, '.vhdr', '');
    % load rawdata -> pop_biosig toolbox
    % EEG = pop_biosig([PATHIN, name{s},'.vhdr']);

    % load rawdata BrainVision
    EEG = pop_loadbv(PATHIN,[ name{s} ,'.vhdr']);

    % Load electrode file
    EEG=pop_chanedit(EEG, 'lookup',chan_file,'load',{chan_file 'filetype'
'autodetect'});
    EEG.comments=''
    EEG.setname = [subj{s}];
    EEG = pop_saveset(EEG, [EEG.setname,'.set'], PATHOUT);
```

```
end
cd(fullfile(MAINPATH, 'scripts', filesep));
% end of script
```

5.2 Running ICA: ana01_ICA.m

```
%%%%%%%%%%%%%%%%%%%%%%%%%%%%%%%%%%%%%%%%%%%%%%%%%%%%%%%%%%%%%%%%%%%%%%%%%%%%%%
%%
% ana01_ICA.m
% Script 1: This script runs an independent component analysis (ICA) on the
raw
% data. The following steps are performed within a subject loop:
%       - Read in Data
%       - Filter data from 1-40 Hz for ICA training
%       - Epoch data (dummy epochs) and remove artificial epochs for ICA
%       - Run ICA with the runica algorithm and the additional option of PCA
%       - Save ICA weights to original data
%
%
% Output: EEG set-file with stored ICA weights
%
% Martin Bleichner 11/01/2018
% Maren Stropahl 25/09/2017

%%%%%%%%%%%%%%%%%%%%%%%%%%%%%%%%%%%%%%%%%%%%%%%%%%%%%%%%%%%%%%%%%%%%%%%%%%%%%%
%%

%% Computer-specific DIRECTORIES
clear; close all;
MAINPATH = fileparts(pwd);
PATHIN  = fullfile(MAINPATH,'rawdata',filesep);      % path containing rawdata
PATHOUT = fullfile(MAINPATH, 'data','ana01',filesep);  % path for script
output
% create output folder if it does not exist yet
if ~exist(PATHOUT)
    mkdir(PATHOUT);
end
% ** add eeglab to Matlab path
addpath(fullfile(MAINPATH,'Software','eeglab14_1_1b'))

% locate rawdata-sets
cd(PATHIN)
list=dir('*.set');  % reads all .set files in PATHIN
len=length(list);   % total number of datasets that will be evaluated
subj=cell(1,len);   % create a subject vector

%% PARAMETERS

HP = 1;         % cut-off frequency high-pass filter [Hz] only for ICA
LP = 40;        % cut-off frequency low-pass filter [Hz] only for ICA
SRATE = 250;    % downsample data for ICA
HP_ord = 500;   % high-pass filter order depends on sampling rate
LP_ord = 100;   % low-pass filter order depends on sampling rate
PRUNE = 3;      % artifact rejection threshold in SD (for ICA only)
PCA = 1;        % choose PCA option for ICA
```

```
PCADIMS = 50;   % PCA dimension if PCA option is true
%% prepare data and run ICA

% start eeglab
[ALLEEG EEG CURRENTSET ALLCOM] = eeglab;

% loop over subjects
for s=1:len
    % define subject name (based on set-files in PATHIN)
    subj{s} = strrep(list(s).name, '.set', '');

    % load rawdata (already saved as set file)
    EEG = pop_loadset('filename', [subj{s}, '.set'], 'filepath', PATHIN);
    EEG.setname = [subj{s}, '_dummy_ICA'];

    % apply low pass filter
    EEG = pop_firws(EEG, 'fcutoff', LP, 'ftype', 'lowpass', 'wtype', 'hann' ,
'forder', LP_ord);
    % downsample data - may be an optional step
    EEG = pop_resample(EEG, SRATE);
    % apply high pass filter
    EEG = pop_firws(EEG, 'fcutoff', HP, 'ftype', 'highpass', 'wtype',
'hann',...
        'forder', HP_ord);

    % create dummy events and epoch data to these dummy events
    EEG = eeg_regepochs(EEG, 'recurrence', 1, 'eventtype', '999');
    EEG = eeg_checkset(EEG, 'eventconsistency');

    % remove epochs with artefacts to improve ICA training
    EEG = pop_jointprob(EEG, 1, [1:size(EEG.data,1)], PRUNE, PRUNE, 0, 1, 0);

    % run ICA optional with our without PCA
    % a window will pop-up as soon as ICA starts which allows to interrupt
    % the ICA process. Please only press if you want to cancel the process
    if PCA == 1
        EEG = pop_runica(EEG, 'icatype', 'runica', 'extended', 1, 'pca',...
            PCADIMS);
    else
        EEG = pop_runica(EEG, 'icatype', 'runica', 'extended', 1);
    end

    % store ICA weights in temporary variables
    icawinv = EEG.icawinv;
    icas = EEG.icasphere;
    icaw = EEG.icaweights;

    % load original rawdata
    EEG = pop_loadset('filename', [subj{s}, '.set'], 'filepath', PATHIN);

    % write ICA weights to rawdata
    EEG.icawinv = icawinv;
    EEG.icasphere = icas;
    EEG.icaweights = icaw;
    EEG = eeg_checkset(EEG);
```

```
    % save new dataset with ICA weights
    EEG.setname = [subj{s}, '_ica'];
    EEG = pop_saveset(EEG, [EEG.setname, '.set'], PATHOUT);
end
cd(fullfile(MAINPATH, 'scripts', filesep));
% end of script
```

5.3 Selection and removal of stereotypical artefacts reflected in ICA components using the semi-automatic CORRMAP algorithm: ana02_corrmap.m

```
%%%%%%%%%%%%%%%%%%%%%%%%%%%%%%%%%%%%%%%%%%%%%%%%%%%%%%%%%%%%%%%%%%%%%%%%%%%%%%
%%
% ana02_corrmap.m
%  Script 2: select ICA components with CORRMAP algorithm
%       1. save all ICA components for each subject as a PNG graphic for
%           inspection of ICA components. Use these figures to select a
template
%           component manually for each artifact
%       2. create study with all datasets containing ICA weights (ana01)
%       3. run CORRMAP to find ICA components based on templates for each
%           subject: heartbeat, eye blink, lateral eye movements
%       4. save components in MAT-file
%           reload data file with saved ICA weights
%           store selected components as EEG.badcomps in each dataset
%           remove selected components
%           save new dataset in PATHOUT with rejected components
%
%
% Output: EEG set-file pruned with ICA
%         MAT-file with saved components (output from CORRMAP)
% Martin Bleichner 11/01/2018
% Maren Stropahl 25/09/2017
%%%%%%%%%%%%%%%%%%%%%%%%%%%%%%%%%%%%%%%%%%%%%%%%%%%%%%%%%%%%%%%%%%%%%%%%%%%%%%
%%

%% Computer-specific DIRECTORIES

clear; close all;

MAINPATH = fileparts(pwd);
PATHIN  = fullfile(MAINPATH,'data','ana01',filesep);      % path containing
rawdata
PATHOUT = fullfile(MAINPATH, 'data','ana02',filesep);  % path for script
output
% create output folder if it does not exist yet
if ~exist(PATHOUT)
    mkdir(PATHOUT);
end
% ** add eeglab to Matlab path
addpath(fullfile(MAINPATH,'Software','eeglab14_1_1b'))

% locate datasets containing ICA weights
cd(PATHIN)
list=dir('*.set');  % reads all .set files in PATHIN
len=length(list);   % total number of datasets that will be evaluated
subj=cell(1,len);   % create a subject vector
```

```
% start eeglab
[ALLEEG EEG CURRENTSET ALLCOM] = eeglab;

%% 1. plot and save all ICA components for later manual selection of template
% components

for s = 1:len % for each subject
    % load dataset
    subj{s}= strrep(list(s).name, '.set', '');
    EEG = pop_loadset('filename', [subj{s}, '.set'], 'filepath', PATHIN);

    % plot ICA components for each subject and save figure as PNG graphic for
    % later inspections
    pop_topoplot(EEG, 0, [1:size(EEG.icawinv,2)], [subj{s}], 0,
'electrodes',...
        'off');
    filename = strrep(subj{s}, '_ica_comps', '');
    saveas(gcf,[PATHIN, filename], 'png');
    close;
end

%% 2. create STUDY with all datasets

% STUDY parameters
studyname = 'ICA_Comps';
% initialize STUDY index
index = 1;
STUDY = []; CURRENTSTUDY = 0; ALLEEG=[]; EEG=[]; CURRENTSET=[];

% set memory options to allow to open more than one dataset:
% in pop_editoptions set 'option_storedisk', 1 or use eeglab GUI
for s = 1:len % for each subject
    % load dataset in study
    subj{s}= strrep(list(s).name, '.set', '');
    dataset = [PATHIN, subj{s}, '.set'];
    [STUDY ALLEEG] = std_editset(STUDY, ALLEEG, 'name', studyname,...
        'commands',{{'index' index 'load' dataset 'subject' subj{s}}},...
        'updatedat', 'off', 'savedat', 'off', 'filename', [PATHIN,
studyname]);
    index = index + 1;

end

CURRENTSTUDY = 1; EEG = ALLEEG; CURRENTSET = [1:length(EEG)];
STUDY.design = [];
[STUDY, ALLEEG] = std_checkset(STUDY, ALLEEG);

% optional: save study
% [STUDY EEG] = pop_savestudy( STUDY, EEG, 'filename',[studyname,
'.study'],...
% 'filepath',PATHIN);

eeglab redraw

%% 3. find ICA components with CORRMAP
```

```
% watch out: semi-automatic process: TEMPLATE COMPONENT has to be selected
% manually! for help see: pop_corrmap.m
% cf. supplementary material for selected components

% optional load study file if saved earlier and if script was not running
% successively
% [STUDY ALLEEG] = pop_loadstudy('filename', [studyname, '.study'],...
% 'filepath', PATHIN);
% CURRENTSTUDY = 1; EEG = ALLEEG; CURRENTSET = [1:length(EEG)];
% [STUDY, ALLEEG] = std_checkset(STUDY, ALLEEG);

% defintion of template datasets and components for each of the three
% components that should be used by the semi-automatic corrmap algorithm
blink_set = 8;     % identified set for the template eye blink component
blink_comp = 1;    % identified template component included in eye_set
heart_set = 8;     % identified set for the template heart beat  component
heart_comp = 10;   % identified template component included in heart_set
eyemov_set = 8;    % identified set for the template eye movement component
eyemov_comp = 17;  % identified template component included in eye_set

% use corrmap to find components in each dataset (data is accessed through
study)
% 1. eye blinks
[CORRMAP, STUDY, ALLEEG] = pop_corrmap(STUDY, ALLEEG, blink_set,
blink_comp,...
    'th', '0.8', 'ics', 3, 'pl', '2nd', 'title', 'plot', 'clname', '', ...
    'badcomps', 'no', 'resetclusters', 'off');
eyeblink = [CORRMAP.output.sets{2} CORRMAP.output.ics{2}];
Corr_eye = CORRMAP;

% 2. heartbeats
[CORRMAP, STUDY, ALLEEG] = pop_corrmap(STUDY, ALLEEG, heart_set,
heart_comp,...
    'th', '0.8', 'ics', 3, 'pl', '2nd', 'title', 'plot', 'clname', '', ...
    'badcomps', 'no', 'resetclusters', 'off');
heartbeat = [CORRMAP.output.sets{2} CORRMAP.output.ics{2}];
Corr_heart = CORRMAP;

% 3. lateral eye movements
[CORRMAP, STUDY, ALLEEG] = pop_corrmap(STUDY,ALLEEG, eyemov_set,
eyemov_comp,...
    'th', '0.8', 'ics', 3, 'pl', '2nd', 'title', 'plot', 'clname', '', ...
    'badcomps', 'no', 'resetclusters', 'off');
eyemovement = [CORRMAP.output.sets{2} CORRMAP.output.ics{2}];
Corr_lateye = CORRMAP;

% save info about components (optional) as MAT-file
save('components.mat', 'eyeblink', 'heartbeat' , 'eyemovement');
save('corrmap_info.mat', 'Corr_eye',  'Corr_heart', 'Corr_lateye');

%% 4. remove components from original dataset

% optional:
% load('components.mat')

% find components marked for each subject, store components in EEG.badcomps
```

```
% and remove selected components from dataset
for s = 1:len
    % load dataset with ICA weights again
    subj{s}= strrep(list(s).name, '.set', '');
    EEG = pop_loadset('filename', [subj{s}, '.set'], 'filepath', PATHIN);

    % find selected components for each subject and save as badcomps
    eye = eyeblink(find(eyeblink(:,1) == s), 2)';
    heart = heartbeat(find(heartbeat(:,1) == s), 2)';
    eyemov = eyemovement(find(eyemovement(:,1) == s), 2)';

    % store all components in EEG structure
    EEG.badcomps = [eye heart eyemov];

    % plot components again, this time with note about badcomps and save as
    % PNG in PATHOUT folder
    pop_topoplot(EEG, 0, [1:size(EEG.icawinv,2)], [subj{s}, ...
        ' removed components: ', num2str(EEG.badcomps)], [6 10], 0, ...
        'electrodes', 'off');
    filename = strrep(subj{s}, '_ica', '_badcomps');
    saveas(gcf, [PATHOUT, filename], 'png');
    close

    % remove selected components from dataset
    EEG = pop_subcomp(EEG, [EEG.badcomps], 0);
    EEG = eeg_checkset(EEG);

    % save ICA cleaned dataset in ana02
    EEG.setname = [subj{s}, '_cleaned'];
    EEG = pop_saveset( EEG, 'filename', [EEG.setname, '.set'], 'filepath',...
        PATHOUT);
end
cd(fullfile(MAINPATH, 'scripts', filesep));

% end of script
```

5.4 Pre-processing of EEG data: ana03_preprocessing.m

```
%%%%%%%%%%%%%%%%%%%%%%%%%%%%%%%%%%%%%%%%%%%%%%%%%%%%%%%%%%%%%%%%%%%%%%%%%%%%%%
%%
% ana03_preprocessing.m
% Script 3: load ICA cleaned data from ana02
%           filter data based on your experimental design
%           epoch data around your experimental events
%           reject contaminated epochs
%           save pre-processed data in PATHOUT
%
%
% Output: pre-processed EEG set-file cleaned, filtered and epoched
% Martin Bleichner 11/01/2018
% Maren Stropahl 25/09/2017
%%%%%%%%%%%%%%%%%%%%%%%%%%%%%%%%%%%%%%%%%%%%%%%%%%%%%%%%%%%%%%%%%%%%%%%%%%%%%%
%%

%% DIRECTORIES
```

```
clear; close all;
MAINPATH = fileparts(pwd);
PATHIN  = fullfile(MAINPATH,'data','ana02',filesep);      % path containing
rawdata
PATHOUT = fullfile(MAINPATH, 'data','ana03',filesep);  % path for script
output

% create output folder if it does not exist yet
if ~exist(PATHOUT)
    mkdir(PATHOUT);
end
% ** add eeglab to Matlab path
addpath(fullfile(MAINPATH,'Software','eeglab14_1_1b'))


% locate datasets
cd(PATHIN)
list=dir('*.set');  % reads all .set files in PATHIN
len=length(list);   % total number of datasets that will be evaluated
subj=cell(1,len);   % create a subject vector

% start eeglab
[ALLEEG EEG CURRENTSET ALLCOM] = eeglab;

%% PARAMETERS
PRUNE = 4;                      % artifact rejection threshold in SD
EPOCH_ON = -0.2;                % epoch start
EPOCH_OFF =0.8;                 % epoch end
HP_erp = 0.1;                   % cut-off frequency high-pass filter [Hz]
LP_erp = 40;                    % cut-off frequency low-pass filter [Hz]
HP_ord = 500;                   % high-pass filter order depends on sampling rate
LP_ord = 100;                   % low-pass filter order depends on sampling rate
ev = 'S 10';                      % event marker for epoching
fs = 1000;                      % sampling rate of EEG recording
baseline = [EPOCH_ON*fs 0]; % definition of baseline

%% start preprocessing of data after ICA cleaning

for s = 1:len
    %load ICA cleaned dataset
    subj{s} = strrep(list(s).name, '_ica_cleaned.set', '');
    EEG = pop_loadset('filename', [subj{s}, '_ica_cleaned.set'],
'filepath',...
        PATHIN);

    % apply low and high pass filter
    EEG = pop_firws(EEG, 'fcutoff', LP_erp, 'ftype', 'lowpass', 'wtype', ...
        'hann', 'forder', LP_ord);
    EEG = pop_firws(EEG, 'fcutoff', HP_erp, 'ftype', 'highpass', 'wtype', ...
        'hann', 'forder', HP_ord);

    % optional step: remove unnecessary event marker (e.g. here fixation
cross)
    % find all events (but S 10 which is the stimulus onset) and save
    % position of EEG.event
    c=1;
```

```
    idx = [];
    for e = 1 : length(EEG.event)
        if ~ strcmp(EEG.event(e).type,ev)
            idx(c) = e;
            c=c+1;
        end
    end
    % remove all events which are not the stimulus onset (here event marker
10)
    EEG = pop_editeventvals(EEG,'delete',idx);
    EEG = eeg_checkset(EEG);

    % epoching and baseline
    EEG = pop_epoch(EEG, {ev}, [EPOCH_ON EPOCH_OFF], 'newname',
EEG.setname,...
        'epochinfo', 'yes');
    EEG = pop_rmbase(EEG, baseline);

    % artifact rejection based on joint probability
    % reject artifacts with joint prob. > PRUNE (SD)
    % optional: store the indices of epoch that are going to be rejected
    % otherwise reject epochs immediately:
    % EEG = pop_jointprob(EEG, 1, [1:EEG.nbchan], PRUNE, PRUNE, 0, 1 , 0);
    EEG = pop_jointprob(EEG, 1, [1:EEG.nbchan], PRUNE, PRUNE, 0, 0 , 0);
    EEG = eeg_rejsuperpose( EEG, 1, 1, 1, 1, 1, 1, 1, 1);
    rej_ep(s) = {find(EEG.reject.rejglobal == 1)};
    EEG = pop_rejepoch( EEG, EEG.reject.rejglobal ,0);

    % optional: get indices of ICA components that were rejected:
    comps(s) = {EEG.badcomps};

    % save pruned and epoched data set
    EEG = eeg_checkset(EEG);
    EEG.setname = [subj{s}, '_ep_ar'];
    EEG = pop_saveset(EEG, [EEG.setname, '.set'], PATHOUT);

end
% optional: save rejected epochs per subject and ICA components that were
% rejected
save([PATHOUT 'info'], 'rej_ep','comps' )

%% All ten preprocessed datasets are stored in a EEGLAB STUDY structure
% This STUDY can then be loaded into EEGLAB for further GUI based analysis
% such as plotting time courses and topographies.
cd(PATHOUT)
list=dir('*.set');  % reads all .set files in PATHIN
len=length(list);   % total number of datasets that will be evaluated
subj=cell(1,len);   % create a subject vector


% STUDY parameters
studyname = 'sensor_level_ERP';
% initialize STUDY index
index = 1;
STUDY = []; CURRENTSTUDY = 0; ALLEEG=[]; EEG=[]; CURRENTSET=[];
```

```
% set memory options to allow to open more than one dataset:
% in pop_editoptions set 'option_storedisk', 1 or use eeglab GUI
for s = 1:len % for each subject
    % load dataset in study
    subj{s}= strrep(list(s).name, '.set', '');
    dataset = [PATHOUT, subj{s}, '.set'];
    [STUDY ALLEEG] = std_editset(STUDY, ALLEEG, 'name', studyname,...
        'commands',{{'index' index 'load' dataset 'subject' subj{s}}},...
        'updatedat', 'off', 'savedat', 'off', 'filename', [PATHOUT,
studyname]);
    index = index + 1;

end

CURRENTSTUDY = 1; EEG = ALLEEG; CURRENTSET = [1:length(EEG)];
STUDY.design = [];
[STUDY, ALLEEG] = std_checkset(STUDY, ALLEEG);


 [STUDY EEG] = pop_savestudy( STUDY, EEG, 'filename',[studyname, '.study'],...
 'filepath',PATHOUT);

cd(fullfile(MAINPATH, 'scripts', filesep));
% end of script
```

5.5 Estimating the source activation of the auditory cortex with brainstorm: ana04_brainstorm.m

```
%%%%%%%%%%%%%%%%%%%%%%%%%%%%%%%%%%%%%%%%%%%%%%%%%%%%%%%%%%%%%%%%%%%%%%%%%%%%%%
%%
% ana04_brainstorm.m
%   before starting the script: create database in brainstorm according to
%   the tutorial (cf. Tutorial 1: Create new protocol
%   http://neuroimage.usc.edu/brainstorm/Tutorials/CreateProtocol).
%   Settings for the protocol should be set to
%   "Default Anatomy: Yes, use protocol's default anatomy"
%   and "Default channel file: Yes, use one channel file per subject"
%   -> see Suppl. Fig. 3
%   Brainstorm database must be opened to run this script
%
%   **Steps below are generated with the brainstorm pipeline editor and
adapted
%   for a group analysis. Parameters are set to brainstorm default.
%   Comments were generated by brainstorm software. For further information
%   concerning individual parameter settings, please refer to the brainstorm
%   tutorials 15-23/28 (http://neuroimage.usc.edu/brainstorm/Tutorials)**
%
% Script 4:
%             1. import pre-processed EEG data (output of script
%             ana03_preprocessing.m
%               set channel file (location of electrodes based on used
%               EEG-cap)
%               compute noise covariance
%               average all trials per subject
%               compute Head model (Boundary-Element-Model, BEM)
%               estimate sources
%               save report
%             2. extract time series of modelled source activity for
%               region-of-interest (scout)
%             3. export the time series of scouts to matlab and save
%
%
% Output: source estimates saved in brainstorm GUI and in PATHOUT
% Martin Bleichner 11/01/2018
% Maren Stropahl 25/09/2017
%%%%%%%%%%%%%%%%%%%%%%%%%%%%%%%%%%%%%%%%%%%%%%%%%%%%%%%%%%%%%%%%%%%%%%%%%%%%%%
%%

%% Directories
clear;
close all;

MAINPATH = fileparts(pwd);
PATHIN  = fullfile(MAINPATH,'data','ana03',filesep);      % path containing
rawdata
PATHOUT = fullfile(MAINPATH, 'data','ana04',filesep);  % path for script
output
% create output folder if it does not exist yet
if ~exist(PATHOUT)
    mkdir(PATHOUT);
end
```

```
% ** set path to your current brainstorm protocol. If you have your
% previously created brainstorm database, please make sure to carefully
% adapt this path
Path_bs = fullfile(MAINPATH,'brainstorm_db','EEG_AEPs','data',filesep);

% ** set the folder with your electrode position file
elec_file = fullfile(MAINPATH,'config','elec_file.xyz');

% specific brainstorm variable used for all processes
sFiles = [];

%%%%%%%%%%%%%%%%%%%%%%%%%%%%%%%%%%%%%%%%%%%%%%%%%%%%%%%%%%%%%%%%%%%%%%%%%%%%%
%%
%% 1. Import and process data with brainstorm %%
%%%%%%%%%%%%%%%%%%%%%%%%%%%%%%%%%%%%%%%%%%%%%%%%%%%%%%%%%%%%%%%%%%%%%%%%%%%%%
%%

% locate pre-processed datasets
cd(PATHIN)
list=dir('*.set');  % reads all .set files in that path
len=length(list);   % total number of datasets that will be evaluated

for s = 1:len
    subj{s} = strrep(list(s).name, list(s).name(4:end), '');
    % start a new report
    bst_report('Start', sFiles);

    % process: Import MEG/EEG: Existing epochs
    sFiles = bst_process('CallProcess', 'process_import_data_epoch', ...
        sFiles, [], ...
        'subjectname',  subj{s}, ...
        'condition',    '', ...
        'datafile',     {{[PATHIN list(s).name ]}, 'EEG-EEGLAB'}, ...
        'iepochs',      [], ...
        'eventtypes',   'type', ...
        'createcond',   1, ...
        'channelalign', 0, ...
        'usectfcomp',   0, ...
        'usessp',       0, ...
        'freq',         [], ...
        'baseline',     []);


    % process: Set channel file
    sFiles = bst_process(...
        'CallProcess', 'process_import_channel', ...
        sFiles, [], ...
        'channelfile', {elec_file, 'EEGLAB'}, ...
        'usedefault', 1, ...
        'channelalign', 0);

    % process: Compute covariance (noise or data)
    sFiles = bst_process('CallProcess', 'process_noisecov', sFiles, [], ...
        'baseline',       [-0.1, -0.002], ...
        'datatimewindow', [], ...
        'sensortypes',    '', ...
```

```
        'target',         1, ...
        'dcoffset',       1, ...
        'identity',       0, ...
        'copycond',       0, ...
        'copysubj',       0, ...
        'replacefile',    1);


    % process: Average: Everything (*what is averaged depends on your
    % experimental conditions)
    sFiles = bst_process('CallProcess', 'process_average', sFiles, [], ...
        'avgtype',    1, ...  % Everything
        'avg_func',   1, ...  % Arithmetic average:  mean(x)
        'weighted',   0, ...
        'keepevents', 0);

    % process: Compute head model (as we use default anatomy, we only need
    % to compute the head model for the 1st subject and we can copy the
    % information to all other subjects, mainly to save some computation
    % time
    if s == 1
        sFiles = bst_process('CallProcess', 'process_headmodel', sFiles, [],
...
            'Comment',     '', ...
            'sourcespace', 1, ...  % Cortex surface
            'volumegrid',  [], ...
            'meg',         2, ...  % Single sphere
            'eeg',         3, ...  % OpenMEEG BEM
            'ecog',        2, ...  % OpenMEEG BEM
            'seeg',        2, ...  % OpenMEEG BEM
            'openmeeg',    struct(...
            'BemFiles',     {{}}, ...
            'BemNames',     {{'Scalp', 'Skull', 'Brain'}}, ...
            'BemCond',      [1, 0.0125, 1], ...
            'BemSelect',    [1, 1, 1], ...
            'isAdjoint',    0, ...
            'isAdaptative', 1, ...
            'isSplit',      0, ...
            'SplitLength',  4000));
    else
        % get the subject for which you have computed the head model
        srcFiles = [Path_bs, list(1).name(1:3), ...
            filesep '@default_study' filesep 'headmodel_surf_openmeeg.mat'];
        % choose the destination folder for the current subject
        StudyFileDest = [Path_bs, list(s).name(1:3), filesep '@default_study'
filesep];
        % copy the head model from subject 1 to the current subject
        copyfile(srcFiles, StudyFileDest, 'f');
        % reload database (might take a while, depending on the size of the
        % current database)
        db_reload_conditions( s )
    end


    % process: Compute sources (2016)
    sFiles = bst_process('CallProcess', 'process_inverse_2016', sFiles, [],
...
```

```
        'output',  2, ...  % Kernel only: one per file
        'inverse', struct(...
        'Comment',         'dSPM: EEG', ...
        'InverseMethod',   'minnorm', ...
        'InverseMeasure',  'dspm', ...
        'SourceOrient',    {{'fixed'}}, ...
        'Loose',           0.2, ...
        'UseDepth',        1, ...
        'WeightExp',       0.5, ...
        'WeightLimit',     10, ...
        'NoiseMethod',     'reg', ...
        'NoiseReg',        0.1, ...
        'SnrMethod',       'fixed', ...
        'SnrRms',          1e-06, ...
        'SnrFixed',        3, ...
        'ComputeKernel',   1, ...
        'DataTypes',       {{'EEG'}}));

    % save and display report
    ReportFile = bst_report('Save', sFiles);
    bst_report('Open', ReportFile);

    % clear brainstorm variable sFiles
    sFiles=[];

end

%%%%%%%%%%%%%%%%%%%%%%%%%%%%%%%%%%%%%%%%%%%%%%%%%%%%%%%%%%%%%%%%%%%%%%%%%%%%%%%%
%%
%%  2. Extract time series of modeled source activity for pre-defined scout
%%%%%%%%%%%%%%%%%%%%%%%%%%%%%%%%%%%%%%%%%%%%%%%%%%%%%%%%%%%%%%%%%%%%%%%%%%%%%%%%
%%
% change directory to current brainstorm database
cd(Path_bs)
% select all subject folders
list=dir('s*');
len=length(list);
subj = {list.name};

for s=1:len
    % locate all source file of the current subject in the bs database
    subj{s} = list(s).name;
    results_files=dir([Path_bs subj{s} filesep subj{s} '_ep_ar' filesep
'results*']);
    % combine path and file name
    source_file = [subj{s} filesep subj{s} '_ep_ar' filesep
results_files.name];

    % Start a new report
    bst_report('Start', sFiles);

    % Process: extract scouts time series: 'S_temporal_transverse R'
    sFiles = bst_process('CallProcess', 'process_extract_scout', ...
        source_file, [], ...
        'timewindow',     [], ...
        'scouts',         {'Destrieux', {'S_temporal_transverse R'}}, ...
        'scoutfunc',      1, ...
```

```
        'isflip',         1, ...
        'isnorm',         0, ...
        'concatenate',    1, ...
        'save',           1, ...
        'addrowcomment',  1, ...
        'addfilecomment', 1);


    % Process: extract scouts time series: 'S_temporal_transverse L'
    sFiles = bst_process('CallProcess', 'process_extract_scout', ...
        source_file, [], ...
        'timewindow',     [], ...
        'scouts',         {'Destrieux', {'S_temporal_transverse L'}}, ...
        'scoutfunc',      1, ...
        'isflip',         1, ...
        'isnorm',         0, ...
        'concatenate',    1, ...
        'save',           1, ...
        'addrowcomment',  1, ...
        'addfilecomment', 1);

    % Save and display report
    ReportFile = bst_report('Save', sFiles);
    bst_report('Open', ReportFile);

end

%%%%%%%%%%%%%%%%%%%%%%%%%%%%%%%%%%%%%%%%%%%%%%%%%%%%%%%%%%%%%%%%%%%%%%%%%%%%%%%
%%
%% 3. export time series of scouts to matlab workspace and save to MAT-file
%%%%%%%%%%%%%%%%%%%%%%%%%%%%%%%%%%%%%%%%%%%%%%%%%%%%%%%%%%%%%%%%%%%%%%%%%%%%%%%
%%

% change directory to current brainstorm database
cd(Path_bs)
% select all subject folders
list=dir('s*');
len=length(list);
subj=[];
subj = {list.name};

sFiles = [];

% name of scouts of which time series should be saved
% here: AC_R: right auditory cortex; AC_L: left auditory cortex
scout = {'AC_R', 'AC_L'};

for s=1:len

    % locate path of source results file
    files=dir([Path_bs, subj{s}, filesep, subj{s}, '_ep_ar', filesep,
'matrix_scout_*']);
    sFiles=strcat({Path_bs}, subj{s}, filesep, subj{s}, '_ep_ar', filesep,
{files(:).name});

    % export to matlab workspace and save
```

```
    for i= 1:length(sFiles)
        export_matlab(sFiles(i), [subj{s}, '_', scout{i}])
        save([PATHOUT,subj{s}, '_', scout{i}, '.mat'], [subj{s},'_',scout{i}])
    end

end
cd(fullfile(MAINPATH, 'scripts', filesep));

% end of script
```

5.6 Advanced brainstorm processing including time-frequency decomposition: ana04_brainstorm_TF.m

```
%%%%%%%%%%%%%%%%%%%%%%%%%%%%%%%%%%%%%%%%%%%%%%%%%%%%%%%%%%%%%%%%%%%%%%%%%%%%%%
%%
% ana04_brainstorm_TF.m
%   before starting the script: create database in brainstorm according to
%   the tutorial (cf. Tutorial 1: Create new protocol
%   http://neuroimage.usc.edu/brainstorm/Tutorials/CreateProtocol).
%   Settings for the protocol should be set to
%   "Default Anatomy: Yes, use protocol's default anatomy"
%   and "Default channel file: Yes, use one channel file per subject"
%   -> see Suppl. Fig. 1
%   Brainstorm database must be opened to run this script
%
%   **Steps below are generated with the brainstorm pipeline editor and
adapted
%   for a group analysis. Parameters are set to brainstorm default.
%   Comments were generated by brainstorm software. For further information
%   concerning individual parameter settings, please refer to the brainstorm
%   tutorials 15-23/28 (http://neuroimage.usc.edu/brainstorm/Tutorials)**
%
% Script 4:
%           1.  import pre-processed EEG data
%               (location of electrodes based on used
%               EEG-cap)
%               compute noise covariance
%               compute Head model (Boundary-Element-Model, BEM)
%               estimate sources for each single trial
%               compute time-frequency decomposition of single-trial source
%               estimate for a selected cluster of scouts (here auditory and
%               occipital scouts, just for demonstration)
%               save report

% Make sure to adapt all paths to the necessary locations on your computer
%
% Output: Source estimates saved in brainstorm GUI and in PATHOUT
% Martin Bleichner 11/01/2018
% Maren Stropahl 25/09/2017
%%%%%%%%%%%%%%%%%%%%%%%%%%%%%%%%%%%%%%%%%%%%%%%%%%%%%%%%%%%%%%%%%%%%%%%%%%%%%%
%%

%% Directories
clear;
close all;
```

```
MAINPATH = fileparts(pwd);
PATHIN  = fullfile(MAINPATH,'data','ana03',filesep);      % path containing
rawdata
PATHOUT = fullfile(MAINPATH, 'data','ana04',filesep);  % path for script
output
% create output folder if it does not exist yet
if ~exist(PATHOUT)
    mkdir(PATHOUT);
end

% ** set path to your current brainstorm database. If you have your
% previously created brainstorm database, please make sure to carefully
% adapt this path
Path_bs = fullfile(MAINPATH,'brainstorm_db','EEG_AEPs','data',filesep);

% ** set the folder with your electrode position file
elec_file = fullfile(MAINPATH,'config','elec_file.xyz')

% specific brainstorm variable used for all processes
sFiles = [];

%%%%%%%%%%%%%%%%%%%%%%%%%%%%%%%%%%%%%%%%%%%%%%%%%%%%%%%%%%%%%%%%%%%%%%%%%%%%%%%%
%%
%%%%%%%%%%%%%%%%%%%%%%%%%%%%%%%%%%%%%%%%%%%%%%%%%%%%%%%%%%%%%%%%%%%%%%%%%%%%%%%%
%%
%% 1. Import and process data
%%%%%%%%%%%%%%%%%%%%%%%%%%%%%%%%%%%%%%%%%%%%%%%%%%%%%%%%%%%%%%%%%%%%%%%%%%%%%%%%
%%

% locate pre-processed datasets
cd(PATHIN)
list=dir('*.set');  % reads all .set files in that path
len=length(list);   % total number of datasets that will be evaluated

for s = 1:len
    subj{s} = strrep(list(s).name, list(s).name(4:end), '');
    % start a new report
    bst_report('Start', sFiles);

    % process: Import MEG/EEG: Existing epochs
    sFiles = bst_process('CallProcess', 'process_import_data_epoch', ...
        sFiles, [], ...
        'subjectname',  subj{s}, ...
        'condition',    '', ...
        'datafile',     {{[PATHIN list(s).name ]}, 'EEG-EEGLAB'}, ...
        'iepochs',      [], ...
        'eventtypes',   'type', ...
        'createcond',   1, ...
        'channelalign', 0, ...
        'usectfcomp',   0, ...
        'usessp',       0, ...
        'freq',         [], ...
        'baseline',     []);


    % process: Set channel file
```

```
    sFiles = bst_process(...
        'CallProcess', 'process_import_channel', ...
        sFiles, [], ...
        'channelfile', {elec_file, 'EEGLAB'}, ...
        'usedefault', 1, ...
        'channelalign', 0);

    % process: Compute covariance (noise or data)
    sFiles = bst_process('CallProcess', 'process_noisecov', sFiles, [], ...
        'baseline',       [-0.1, -0.002], ...
        'datatimewindow', [], ...
        'sensortypes',    '', ...
        'target',         1, ...
        'dcoffset',       1, ...
        'identity',       0, ...
        'copycond',       0, ...
        'copysubj',       0, ...
        'replacefile',    1);


    % process: Compute head model (as we use default anatomy, we only need
    % to compute the head model for the 1st subject and we can copy the
    % information to all other subjects, mainly to save computation time
    if s == 1
        sFiles = bst_process('CallProcess', 'process_headmodel', sFiles, [],
...
            'Comment',      '', ...
            'sourcespace', 1, ...  % Cortex surface
            'volumegrid',  [], ...
            'meg',         2, ...  % Single sphere
            'eeg',         3, ...  % OpenMEEG BEM
            'ecog',        2, ...  % OpenMEEG BEM
            'seeg',        2, ...  % OpenMEEG BEM
            'openmeeg',    struct(...
            'BemFiles',     {{}}, ...
            'BemNames',     {{'Scalp', 'Skull', 'Brain'}}, ...
            'BemCond',      [1, 0.0125, 1], ...
            'BemSelect',    [1, 1, 1], ...
            'isAdjoint',    0, ...
            'isAdaptative', 1, ...
            'isSplit',      0, ...
            'SplitLength',  4000));
    else
        % get the subject for which you have computed the head model
        srcFiles = [Path_bs, list(1).name(1:3), ...
            filesep '@default_study' filesep 'headmodel_surf_openmeeg.mat'];
        % choose the destination folder for the current subject
        StudyFileDest = [Path_bs, list(s).name(1:3), filesep '@default_study'
filesep];
        % copy the head model from subject 1 to the current subject
        copyfile(srcFiles, StudyFileDest, 'f');
        % reload database (might take a while, depending on the size of the
        % current database)
        db_reload_conditions( s )
    end

    % process: Compute sources (2016)
```

```
    sFiles = bst_process('CallProcess', 'process_inverse_2016', sFiles, [],
...
        'output',  2, ...  % Kernel only: one per file
        'inverse', struct(...
        'Comment',        'dSPM: EEG', ...
        'InverseMethod',  'minnorm', ...
        'InverseMeasure', 'dspm', ...
        'SourceOrient',   {{'fixed'}}, ...
        'Loose',          0.2, ...
        'UseDepth',       1, ...
        'WeightExp',      0.5, ...
        'WeightLimit',    10, ...
        'NoiseMethod',    'reg', ...
        'NoiseReg',       0.1, ...
        'SnrMethod',      'fixed', ...
        'SnrRms',         1e-06, ...
        'SnrFixed',       3, ...
        'ComputeKernel',  1, ...
        'DataTypes',      {{'EEG'}}));

    % process: Time-frequency (Morlet wavelets)
    % a cluster of scouts is used for which the TF will be computed
    sFiles = bst_process('CallProcess', 'process_timefreq', sFiles, [], ...
        'clusters',  {'Destrieux', {'Pole_occipital L', 'Pole_occipital R',
...
        'S_temporal_transverse L', 'S_temporal_transverse R'}}, ...
        'scoutfunc', 1, ...  % Mean
        'edit',      struct(...
        'Comment',         'Scouts,Avg,Power,1-60Hz', ...
        'TimeBands',       [], ...
        'Freqs',           [5:1:60], ...
        'MorletFc',        1, ...
        'MorletFwhmTc',    3, ...
        'ClusterFuncTime', 'after', ...
        'Measure',         'power', ...
        'Output',          'average', ...
        'RemoveEvoked',    0, ...
        'SaveKernel',      0), ...
        'normalize', 'none');  %None: Save non-standardized time-frequency
maps

    % save and display report
    ReportFile = bst_report('Save', sFiles);
    bst_report('Open', ReportFile);

    % clear brainstorm variable sFiles
    sFiles=[];

end
cd(fullfile(MAINPATH, 'scripts', filesep));

% end of script
```
